# Effect of antiplatelet therapy after COVID-19 diagnosis: A systematic review with meta-analysis and trial sequential analysis

**DOI:** 10.1371/journal.pone.0297628

**Published:** 2024-02-01

**Authors:** Hong Duo, Mengying Jin, Yanwei Yang, Rewaan Baheti, Yujia Feng, Zirui Fu, Yuyue Jiang, Lanzhuoying Zheng, Jing Wan, Huaqin Pan

**Affiliations:** 1 Zhongnan Hospital of Wuhan University, Institute of Hepatobiliary Diseases of Wuhan University, Transplant Center of Wuhan University, Hubei Key Laboratory of Medical Technology on Transplantation, Wuhan, China; 2 Department of Cardiology, Zhongnan Hospital of Wuhan University, Wuhan, Hubei, China; 3 Department of Critical Care Medicine, Zhongnan Hospital of Wuhan University, Wuhan, China; 4 College of Agriculture and Life Science, University of Wisconsin Madison, Madison, Wisconsin, United States of America; 5 University of California, Santa Barbara/ UC Santa Barbara, Santa Barbara, California, United States of America; 6 Clinical Research Center for Critical Care Medicine of Hubei Province, Wuhan, China; Hamad Medical Corporation, QATAR

## Abstract

**Background:**

Coronavirus disease 2019 (COVID-19) may predispose patients to thrombotic disease in the venous and arterial circulations.

**Methods:**

Based on the current debate on antiplatelet therapy in COVID-19 patients, we performed a systematic review and meta-analysis to investigate the effect of antiplatelet treatments. We searched PubMed, EMBASE, Cochrane Central Register of Controlled Trials, and Web of Science on February 1, 2023, and only included Randomized clinical trials. The study followed PRISMA guidelines and used Random-effects models to estimate the pooled percentage and its 95% CI.

**Results:**

Five unique eligible studies were included, covering 17,950 patients with COVID-19. The result showed no statistically significant difference in the relative risk of all-cause death in antiplatelet therapy versus non-antiplatelet therapy (RR 0.94, 95% CI, 0.83–1.05, *P* = 0.26, I^2^ = 32%). Compared to no antiplatelet therapy, patients who received antiplatelet therapy had a significantly increased relative risk of major bleeding (RR 1.81, 95%CI 1.09–3.00, *P* = 0.02, I^2^ = 16%). The sequential analysis suggests that more RCTs are needed to draw more accurate conclusions. This systematic review and meta-analysis revealed that the use of antiplatelet agents exhibited no significant benefit on all-cause death, and the upper bound of the confidence interval on all-cause death (RR 95% CI, 0.83–1.05) suggested that it was unlikely to be a substantiated harm risk associated with this treatment. However, evidence from all RCTs suggested a high risk of major bleeding in antiplatelet agent treatments.

**Conclusion:**

According to the results of our sequential analysis, there is not enough evidence available to support or negate the use of antiplatelet agents in COVID-19 cases. The results of ongoing and future well-designed, large, randomized clinical trials are needed.

## Introduction

Coronavirus disease 2019 (COVID-19) represents an unprecedented global threat caused by severe acute respiratory syndrome coronavirus 2 (SARS-CoV-2) [1]. COVID-19 may predispose patients to thrombotic disease in both venous and arterial circulations, due to excessive inflammation, platelet activation, endothelial dysfunction, and stasis [2]. Critically ill COVID-19 patients are at an elevated risk of hypercoagulability and increased thrombotic risk [3]. Interactions between activated platelets and neutrophils lead to the breakdown of extracellular matrix proteins and the production of thrombin, which may be associated with disseminated intravascular coagulation (DIC) and a hypercoagulable state [4]. Early antiplatelet drugs may inhibit uncontrolled adhesion, aggregation, and platelet activation, thereby reducing the risk of severe organ dysfunction. On the one hand, in severe COVID-19 patients, especially in patients admitted to the ICU, venous thromboembolism (including extensive deep vein thrombosis and pulmonary embolism) is common and has been reported to occur in 10%-40% of this population [5–8]. To address this issue, low molecular weight heparin can be employed, which is known to reduce the risk of venous thromboembolism (VTE) in medical inpatients and also possesses anti-inflammatory effects [9–11]. According to the American Society of Hematology 2021 guidelines, prophylactic doses of anticoagulation are conditionally recommended for acute and critically ill COVID-19 patients rather than higher doses of anticoagulation [12]. On the other hand, arterial thrombosis has also been reported, including acute stroke (even in patients younger than 50 years without risk factors) and limb ischemia [13–16]. Even some observational clinical studies have shown a reduction in mortality among COVID-19 patients in the aspirin group compared to the non-aspirin group [17]. Aspirin use in high-risk patients hospitalized with COVID-19 may be associated with reduced mortality [3], but its efficacy in moderately ill patients with COVID-19 has not been adequately studied [18]. Meanwhile, the role of antiplatelet drugs other than aspirin is being investigated [19, 20]. Despite the rationale that early antiplatelet would lower the risk of major organ dysfunction, the effectiveness of this approach remains controversial. In addition, while current anticoagulant therapy has shown effectiveness, the effect of antiplatelet therapy based on anticoagulation is still controversial. While these studies [21, 22] covered attempted research on the effects of antiplatelet therapy, no randomized trials meta-analysis and trial sequential analysis was performed. Therefore, we conducted a systematic review and meta-analysis following the PICO framework to investigate on the effects of antiplatelet treatments, such as aspirin, clopidogrel, and P2Y12 inhibitors, on patients diagnosed with COVID-19. The study population comprised patients with confirmed COVID-19 infection on anticoagulation therapy. The intervention included antiplatelet therapy, while the comparison involved placebo or standard treatments in combination with anticoagulants. The outcomes assessed encompassed mortality, bleeding events, and arterial or venous thrombosis.

## Methods

Our comprehensive study was performed in accordance with the 2020 Preferred Reporting Items for Systematic Reviews and Meta-analyses (PRISMA) reporting guideline (**S1 Table**) [23]. A systematic review protocol to PROSPERO in advance (registration number: CRD42022321234) was developed and registered.

### Data sources, searches, and study selection

Three investigators (HD, MYJ, and YWY) systematically searched PubMed, Embase, Cochrane Central Register of Controlled Trials, and Web of Science databases till February 1, 2023. Searched strategies included “COVID-19 OR COVID19 OR SARS-CoV-2” and “Clopidogrel OR Aspirin OR ticagrelor” (**S2 Table**).

There were no restrictions on sample size or geographical location for included studies. Instead, restrictions on the language used in paper (only English), COVID-19 patients’ ages (>18 years) were set, and the inclusion of patients diagnosed with COVID-19. Studies were included if they compared antiplatelet therapy to no antiplatelet therapy and provided available data on at least one of our primary outcomes (all-cause death). Inclusion and exclusion of PICOS criteria (Population, Intervention, Comparison, Outcome, and Study) (**S3 Table**) was conducted. Two investigators (HD and MYJ) independently screened titles and abstracts to determine suitability based on our baseline outcomes. We also retrieved and analyzed full-text articles for eligibility. Any controversies were resolved by consensus, when necessary, by involving a third reviewer (RWA) until a resolution was reached. The following types of articles were excluded: reviews, editorials, commentaries, conference abstracts, case reports, meeting abstracts, practice guidelines, and protocols.

### Data extraction and risk of bias assessment

Two reviewers (MYJ and YWY) independently extracted relevant data from the item report and supplementary files. Disagreements between the reviewers were resolved by consensus or arbitrated by a third reviewer (HD). The collected data included information on study design, study population, sample size total, age, sex, race, body mass index (BMI), antiplatelet therapy administration, a dose of antiplatelet agents, c-reactive protein, d-dimer, platelet count, follow-up days, respiratory support received, and invasive mechanical ventilation. Two investigators (HD and RWA) independently undertook a methodological quality assessment of the Cochrane risk-of-bias tool 2.0 [24]. Five domains of bias were assessed: randomization process, deviations from intended interventions, missing outcome data, measurement of the outcome, selection of the reported result, and overall bias. The quality of the randomized controlled trial was assessed with the Risk of Bias tool, assigning high risk, low risk, or some concern.

### Quality of evidence

To evaluate the overall quality of evidence, we used the GRADE system. The evaluation involved key domains, including the risk of bias, indirectness, inconsistency, imprecision, and other Considerations. A final level of evidence adjudication for a certain body of evidence was achieved by a qualitative discussion of each GRADE item by two investigators (RWA and MYJ). In case of any disparities during this process, the third investigator (YWY) made the final decision.

### Definition

We assessed six distinct outcomes in our study. The primary efficacy outcome was all-cause death, which we defined as patients who died from various causes during the follow-up period (21 days, 28 days, and 90 days for ACTIV-4a, RECOVERY/PACT, and REMAP-CAP, respectively). The secondary outcomes included major bleeding events, which are defined as including fatal bleeding, symptomatic or clinically manifest bleeding within critical anatomical or organ sites such as intracranial, spinal, ocular, retroperitoneal, intra-articular, intrapericardial bleeding, or bleeding within muscles associated with compartment syndrome. Additionally, these bleeding events may also result in a decrease in hemoglobin of 2 g/dL or more or necessitate the transfusion of 2 units or more of whole blood or red blood cells [25]. Other secondary outcomes were survival to hospital discharge (number of patients who survived and were discharged within 90 days for ACTIV-4a and REMAP-CAP, and within 28 days for RECOVERY and PACT), any thrombotic event (including a composite endpoint including myocardial infarction, ischemic cerebrovascular events, deep vein thrombosis, pulmonary embolism, or systemic arterial thromboembolism diagnosed during hospitalization or leading to in-hospital death), as well as venous thrombotic events (including deep vein thrombosis, pulmonary embolism, and other venous thromboembolism), and arterial thrombotic events (including cerebrovascular events, myocardial infarction, and other arterial thrombotic events).

### Statistical analyses

We extracted the available variables of the defined outcome parameters from the full-text publications and supplementary files, if available. Then we conducted the Mantel-Haenszel test with random effects to perform pooled analyses and carried out random effects models to test consistency to reduce the effect of heterogeneity. We assessed heterogeneity among the studies using both the P-value of the Cochran Q test and the I^2^ statistic, along with their respective 95% confidence intervals. These measures can reflect either genuine heterogeneity or potential bias. The I^2^ statistic ranges from 0% and 100% (with values of 0%-25% and 75%-100% taken to indicate low and considerable heterogeneity, respectively). We performed subgroup analysis by dividing the studies into diverse groups according to baseline characteristics (different interventions) and study heterogeneity. To decrease the bias and eliminate the impact of population heterogeneity and interventions, we classified the data into aspirin and P2Y12 inhibitor groups to avoid bias caused by offsetting the total results. We also performed the trial sequential analysis to test whether the included studies were sufficient to reach a concrete conclusion. In a sensitivity analysis, we sequentially excluded one trial at a time and reanalyzed the data to determine whether any study influenced the results. The results of meta-analyses do not reflect actual effects, possibly due to random error. Furthermore, the NCT02735707 lacks statistical significance, which may be due to a lack of statistical power. Random errors are more likely to occur in meta-analyses with fewer studies or populations included in studies. Trial sequential analysis (TSA) is analogous to the interim analysis in clinical trials. We calculated the estimated sample size for the meta-analysis to be powered for a two-sided type 1 error of 5.0% with a power of 80%, utilizing the O’Brien-Fleming α-spending function. We minimized the type 1 error in the TSA by using a highly stringent criterion of 5% type 1 error and 80% power. TSA was performed using TSA version 0.9. We provided TSA-adjusted CI for sparse data and repeated testing, which we described as trial sequential analysis-adjusted CI. We meticulously reviewed both trial registrations and formally published articles to assess the potential presence of publication bias. Our Meta-Analysis was performed using Review Manager 5.4 (Revman, Te Cochrane Collaboration, Oxford, UK).

## Results

### Search results

**Fig 1:**
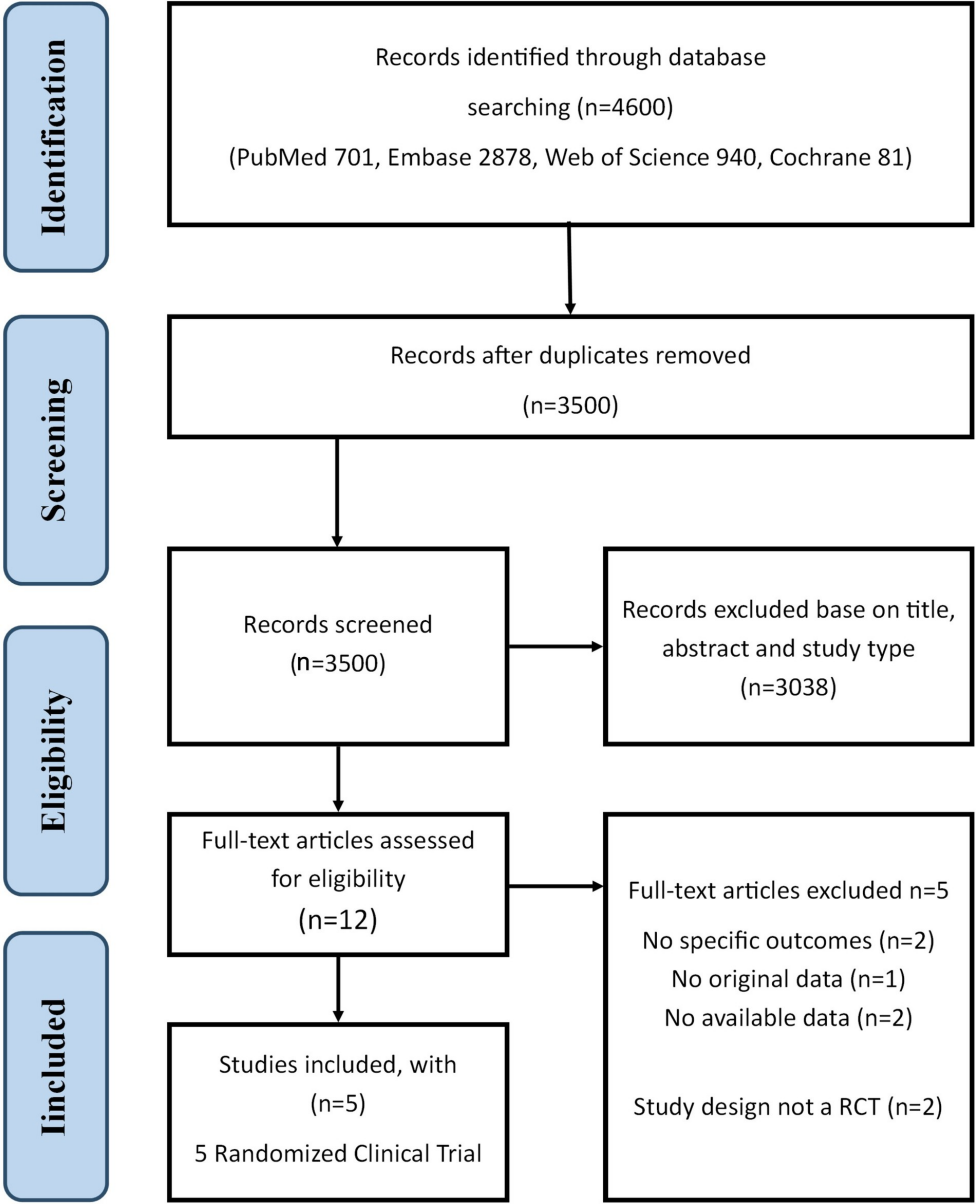
Flow diagram of the literature search and selection. We screened the original literature records through database searching and identified a total of 4600 articles. Twelve articles were assessed for eligibility and retrieved for full-text review after removal of duplicates and screening based on article title, abstract, and type.

Five studies met our inclusion criteria in **Fig 1** [25–29]. Characteristics of the included studies are shown in **Table 1** and **S4 Table**. Primary outcomes and secondary outcomes are shown in **Table 2**. **S5 and S6 Tables** supplement primary outcomes and secondary outcomes. Five studies fulfilled our inclusion criteria of randomized control trials, with a combined population of 17,950 patients. 1549 patients from REMAP-CAP [25] are comparing aspirin and P2Y12 inhibitor versus no antiplatelet therapy in critically ill patients hospitalized for COVID-19; 562 patients from ACTIV-4a [26] comparing P2Y12 inhibitor versus a therapeutic dose of heparin only (usual care) in non-critically ill patients hospitalized for COVID-19; 657 patients from ACTIV-4B [27] comparing aspirin versus apixaban and placebo in symptomatic outpatients with COVID-19; 14892 patients from RECOVERY [28] comparing aspirin versus usual standard of care in patients hospitalized with COVID-19;290 patients from PACT [29] comparing clopidogrel versus no clopidogrel in critically ill patients hospitalized for COVID-19. Four of the five studies [25, 26, 28, 29] were open-label RCTs; only the ACTIV-4B [27] trial was double-blind. ACTIV-4a had the shortest follow-up days (21 days), whereas REMAP-CAP had the longest (90 days). The PROSPERO protocol reports the primary outcomes as “Thrombotic events or death”. Although additional outcomes were not specified in the protocol, we added some outcome measures after weighing the specific clinical benefits of antiplatelet therapy, all of which are available.

**Table 1:** Baseline characteristics.

Study	REMAP-CAP	ACTIV-4a	RECOVERY	ACTIV-4B	PACT
Study design	randomized open-label clinical trial RCT	open-label, bayesian, adaptive randomized clinical Trial RCT	individually randomized, controlled, open-label, platform, investigator-initiated trial RCT	minimal-contact, adaptive, randomized, double-blind, placebo-controlled trial RCT	open-label, blinded end point adjudication, RCT
Study population	non–critically and critically ill patients hospitalized for COVID-19	non–critically ill patients hospitalized for COVID-19	patients hospitalized with COVID-19	symptomatic outpatients with COVID-19	critically ill patients hospitalized for COVID-19
Sample size, n, total	1549	562	14 892	657	290
Antiplatelet therapy, n, total	1020	293	7351	164	150
Control, n, total	529	269	7541	493	140
Age, years, median	57 vs. 57	53.1 vs. 52.3 ^a^	59.2 vs. 59.3	54 vs. 53.67	58 vs. 58
Sex					
Male, n, total	682 vs. 346	169 vs. 160	4570 vs. 4631	69 vs. 200	92 vs.80
Female, n, total	338 vs. 183	124 vs. 109	2781 vs. 2910	95 vs. 293	58 vs.60
Race					
White, n, total	659 vs. 309	167 vs. 152	5474 vs. 5655	119 vs. 375	115 vs. 91
Black, Asian, and minority, n, total	107 vs. 74	75 vs. 76	1176 vs. 1202	23 vs. 64	Not reported
Unknown, n, total	66 vs. 36	27 vs. 18	701 vs. 684	7 vs. 27	Not reported
BMI, kg/m^2^, median	31.5 vs. 31.1	31.4 vs. 31.6	Not reported	29.6 vs. 30.43	34 vs.34
Antiplatelet therapy administration	Aspirin or P2Y12 inhibitors vs. no antiplatelet ^e^	Therapeutic dose of heparin plus P2Y12 inhibitor vs. Therapeutic dose of heparin only ^f^	Aspirin vs. usual care ^g^	Aspirin vs. Apixaban; Placebo ^h^	Clopidogrel vs. No Clopidogrel
Dose of antiplatelet agents	aspirin, 75 to 100mg once daily; clopidogrel, 75mg once daily; ^d^	Therapeutic dose of heparin; Ticagrelor 60 mg twice daily;	150mg aspirin once per day;	81 mg aspirin once daily;	Clopidogrel 300 mg once orally on the day of randomization, followed by 75 mg once daily on subsequent days
Biochemistry					
C-reactive protein, mg/L, median	113.5 vs. 113	93.8 vs. 93.0	88 vs. 91	3.1 vs. 4.23 ^b^	not reported
D-dimer, μg/mL, median	911 vs. 898	1.017 vs. 1.060	475 vs. 489	146 vs. 151.67	807 vs. 902
Platelet count, ×10^9^/L, median	245 vs. 253	211 vs. 217	Not reported	246 vs 245.33	not reported
Follow-up, total, days	90	21^i^	28	30^c^	28
Number of days since symptom onset	not reported	not reported	9 to 9	not reported	not reported
Number of days since hospitalization	not reported	not reported	1 to 2	not reported	not reported

Abbreviations: BMI, Body mass index

^a^ The values are mean

^b^ The values means the numbers of hsCPR

^c^ This means the days of safety follow-up

^d^ Antiplatelet dosing was as follows: aspirin, 75 to 100mg once daily; clopidogrel, 75mg once daily without a loading dose; ticagrelor, 60mg twice daily without a loading dose; prasugrel, a 60-mg loading dose followed by 10mg daily (if aged <75 years and weight ≥60 kg) or 5mg daily (if aged ≥75 years or weight <60 kg)

^e^ All patients with data available (n = 1419) received concurrent thromboprophylaxis according to usual care at the site or were concomitantly enrolled in the platform anticoagulation study. The most frequent concurrent anticoagulant was low-molecular-weight heparin (97.7%), and the most frequent dose was an intermediate dose (59%)

^f^ All patients received thromboprophylaxis. The most frequent concurrent anticoagulant was heparin or low-molecular-weight heparin. These drugs are considered standard of care as anticoagulants. Initial bolus dose determined by sites, encouraging the use of dosing algorithm designed for treatment of VTE.

^G^ 5035 (34%) patients were receiving thromboprophylaxis with higher-dose low molecular weight heparin (LMWH), 8878 (60%) patients were receiving standard-dose LMWH and 979 (7%) patients were not receiving thromboprophylaxis.

^H^ Participants were randomized centrally in a 1:1:1:1 ratio to receive aspirin (81 mg once daily) with matching placebo, prophylactic-dose apixaban (2.5 mg twice daily), apixaban at the therapeutic dose (5 mg twice daily), or placebo twice daily for 45 days, with a 30-day safety follow-up evaluation.

^I^ The number of days with organ support or death over the first 21 days of the index hospitalization, remained high despite treatment with therapeutic dose anticoagulants, particularly in certain subsets, and bleeding risk was < 2%.

^J^ As for Data Processing of REMAP-CAP and ACTIV-4B: continuous variables (Age, BMI, C-Reactive Protein, D-Dimer, Platelet count) should be represented by post-merger mean, but categorical variables (Sex, Race, Previous Diseases, Concomitant therapies) should be represented by the sum of groups.

**Table 2:** Description of primary outcome and secondary outcomes of meta-analysis.

	REMAP-CAP		ACTIV-4a		RECOVERY		ACTIV-4b		PACT	
Intervention	Pooled antiplatelet therapy	Control	P2Y12 inhibitor	Usual care	Aspirin	Usual care	Aspirin	Placebo	Clopidogrel	No clopidogrel
All-cause death, n, total	299/1011(29.6%)	170/521(32.6%)	18/293(6.1%)	11/269(4.1%)	1222/7351(16.6%)	1299/7541(17.2%)	0/144(0%)	0/414(0%)	24/150(16%)	34/140(24.3%)
Survival to hospital discharge, n, total	723/1011(71.5%)	354/521(67.9%)	275/293(93.9%)	258/269(95.9%)	5496/7351(74.8%)	5548/7541(73.6%)	-	-	111/150(74%)	106/140(75.7%)
Any thrombotic event, n, total	112/996(11.2%)	65/513(12.7%)	9/293(3.1%)	5/269(1.9%)	339/7290(4.6%)	396/7457(5.3%)	0	0	17/150(11.3%)	21/140(15%)
Venous thrombotic event, n, total	87/998(8.7%)	56/516(10.9%)	5/293(1.7%)	5/269(1.9%)	321/7290(4.4%)	372/7457(5.0%)	0	0	17/150(11.3%)	21/140(15%)
Arterial thrombotic event, n, total	37/996(3.7%)	12/513(2.3%)	4/293(1.4%)	1/269(0.4%)	27/7290(0.4%)	41/7457(0.5%)	0	0	1/150(0.7%)	0/140(0%)
Major bleeding, n, total	21/1002(2.1%)	2/517(0.4%)	6/293(2.0%)	2/269(0.7%)	115/7290(1.6%)	76/7457(1.0%)	0	0	2/150(1.3%)	2/140(1.4%)
Aggravation of illness, n, total	636/1011(62.9%)	326/521(62.6%)	-	-	1473/6993(21.1%)	1569/716(21.9%)	-	-	-	-

**S7 Table** shows a detailed list of the different intervention regimens and outcomes definitions and a comprehensive overview of each trial. The Supplement provides a summary of patients’ baseline characteristics of included trials.

**Fig 2:**
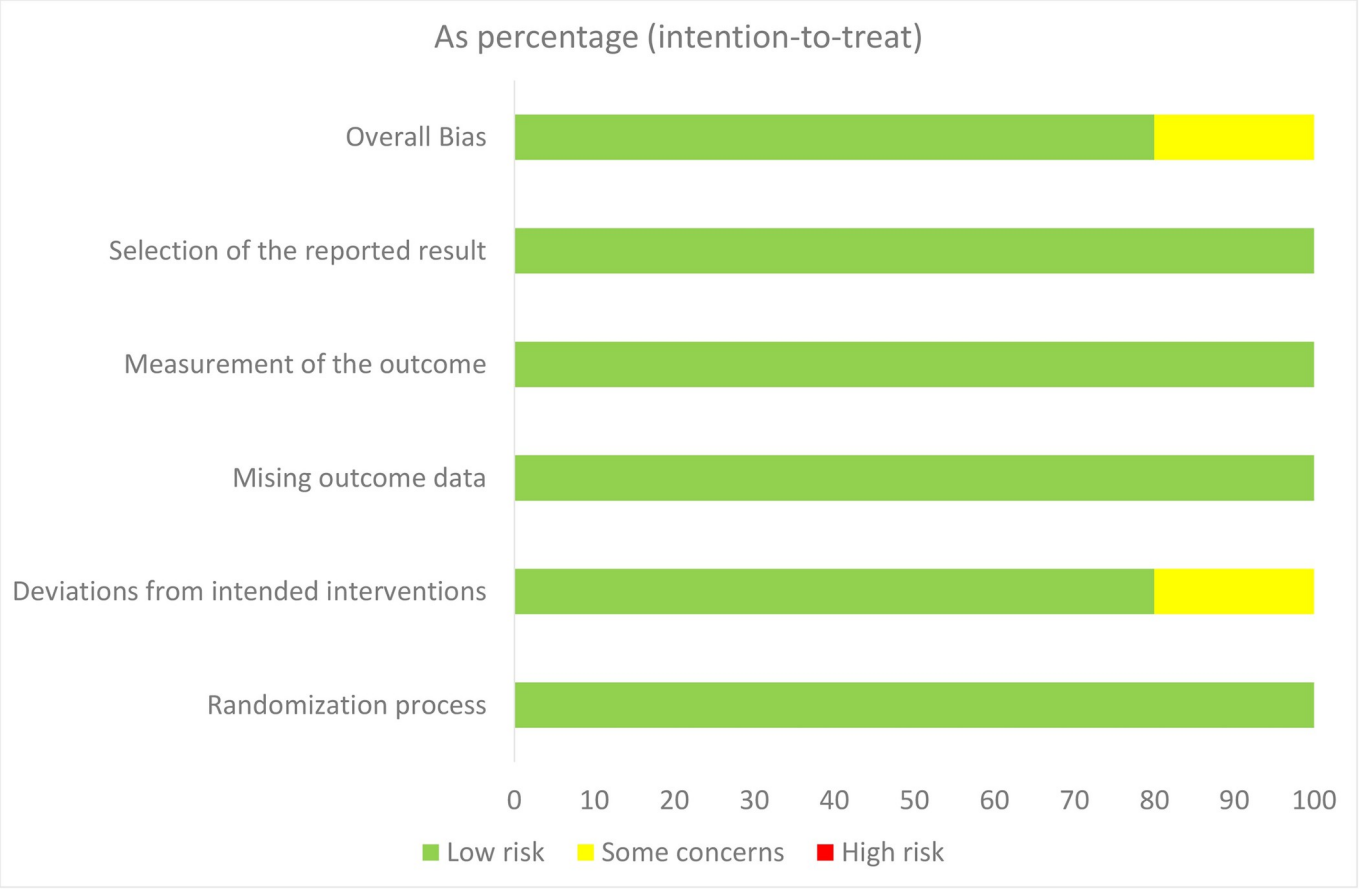
Distribution across studies for each risk of bias.

The Quality assessment of included studies by Risk of bias 2.0 in **S8 Table**. Four studies (ACTIV-4a, ACTIV-4b, REMAP-CAP, and PACT) that estimate the overall risk bias, which focuses on all-cause death, had a low risk of bias, as determined using the Cochrane risk-of-bias tool for randomized trials (RoB2) During the RECOVERY trial, participants and local study staff were not masked to the allocated treatment which led to some concern about the risk of bias in deviations from intended interventions (**Fig 2**). The quality of evidence is in **S9 Table**. A high level of evidence was identified for randomized studies that reported all-cause death, survival to hospital discharge, any thrombotic event, venous thrombotic event, arterial thrombotic event, and major bleeding.

## Primary efficacy outcome

**Fig 3:**
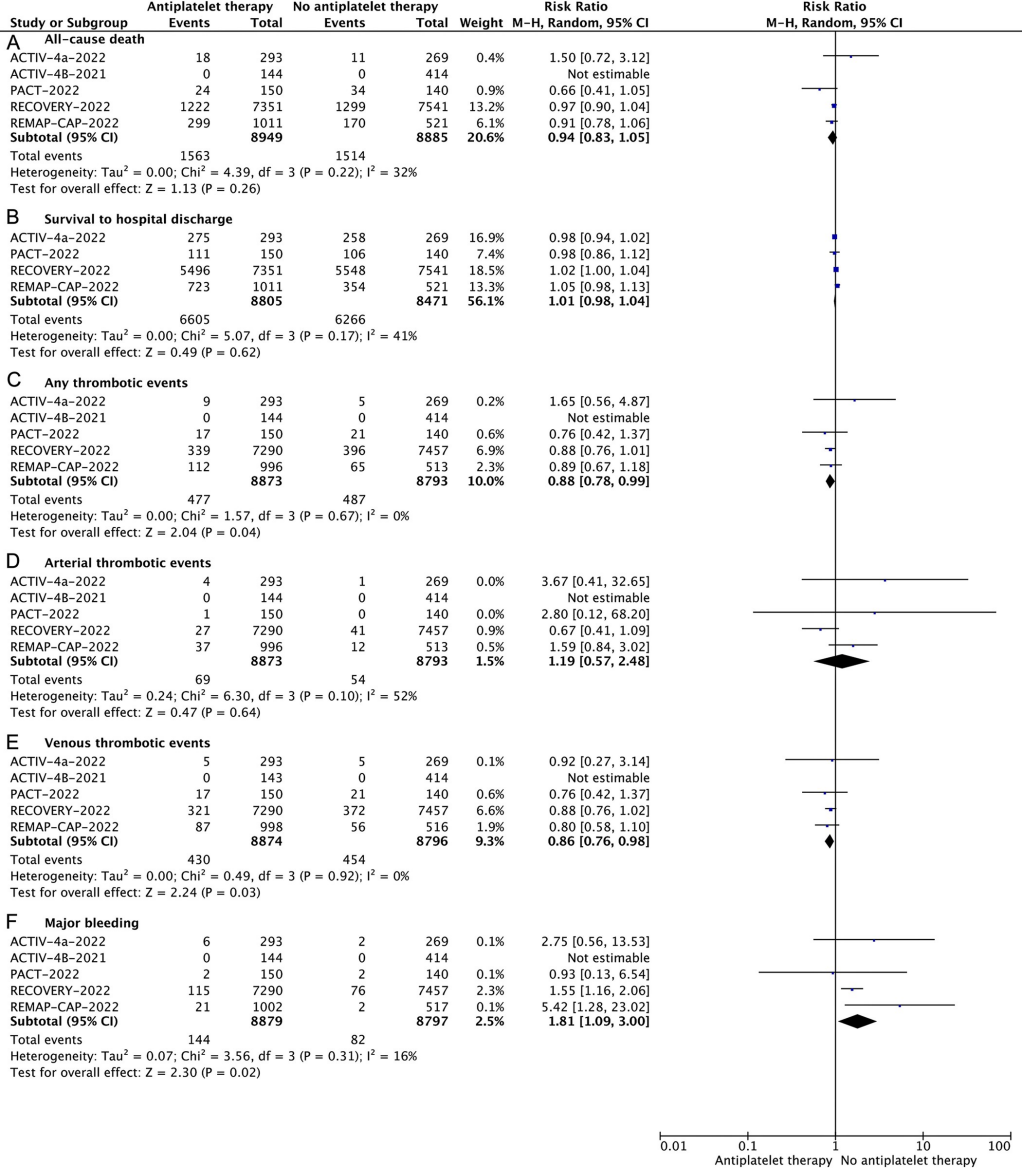
Forest plots depicting risk ratios of all-cause death (A), survival to hospital discharge (B), any thrombotic events(C), venous thrombotic events(D), arterial thrombotic events(E) and major bleeding(F) for comparison between antiplatelet therapy and no antiplatelet therapy. CI, confidence interval.

All five included studies have reported all-cause death. The primary efficacy outcome is all-cause death. Overall, all-cause death was 17.4% (1563/8949) and 17.0% (1514/8885) in patients treated with antiplatelet and no antiplatelet therapy, respectively. ACTIV-4B trial was excluded for symptomatic outpatients with COVID-19, which had no available data for outcome events to calculate relative risk. In our overall meta-analysis, there was no statistically significant difference in the relative risk of all-cause death between antiplatelet therapy and no antiplatelet therapy (RR 0.94, 95% CI, 0.83–1.05, *P* = 0.26, I^2^ = 32%; trial sequential analysis adjusted RR 0.95, CI 0.89–1.02; **Fig 3A and S1 Fig**). In the trial sequential analysis, the Z curve did not cross any of the boundaries. That is, within the set assumptions for confidence and effect size, for the result of all-cause death, this intervention is not clear. This finding, of course, comes with a 20% risk of being a false futile finding (the type II error is 20%).

### Secondary outcomes

The incidence of major bleeding was 1.6% (144/8879) in the antiplatelet therapy and 0.9% (82/8789) in no antiplatelet treatment, respectively. Compared to no antiplatelet therapy, patients who received antiplatelet therapy had significantly increased relative risk of major bleeding (RR 1.69, 95%CI 1.28–2.22, *P* = 0.02, I^2^ = 16%; trial sequential analysis adjusted RR 1.69, CI 1.29–2.22; **Fig 3F and S6 Fig)**. In the trial sequential analysis, the Z-curve crossed the conventional test boundary and trial sequential monitoring boundaries, but the cumulative information did not reach the required information size, indicating that the traditional meta-analysis is robust regardless it does not reach the required information size.

Four studies included the outcome of survival to hospital discharge. The incidence of survival to hospital discharge was 75.0% (6605/8805) with the antiplatelet therapy and 73.9% (6266/8471) with no antiplatelet therapy. Antiplatelet therapy was not statistically significant for survival to hospital discharge compared to no antiplatelet therapy. (RR 1.01, 95% CI, 0.98–1.04, *P* = 0.62, I^2^ = 41%; trial sequential analysis adjusted RR 1.02, CI 1.00–1.03; **Fig 3B and S2 Fig**). In the trial sequential analysis, cumulative information did not reach the required information size, so there may be no statistical difference in survival to discharge outcomes between the intervention and control groups. More trials are needed to confirm the second.

Five studies included the outcome of any thrombotic event. The incidence of any thrombotic event was 5.3% (477/8873) in the antiplatelet therapy and 5.5% (487/8793) in the no antiplatelet therapy, respectively. Compared to no antiplatelet therapy, thrombotic events decreased in patients treated with antiplatelet therapy (RR 0.88, 95%CI 0.78–0.99, *P* = 0.04, I^2^ = 0%; trial sequential analysis adjusted RR 0.89, CI 0.78–1.00 **Fig 3C and S3 Fig**). In the trial sequential analysis, the cumulative information did not reach the required information size, but the Z-curve crossed the conventional test boundary, indicating it may yield false-positive conclusions. Meanwhile, the incidence of arterial thrombotic events in antiplatelet therapy and no antiplatelet therapy, respectively, resulting in no significant differences between regimens (RR 1.19, 95%CI 0.57–2.48, *P* = 0.64, I^2^ = 52%; trial sequential analysis adjusted RR 1.00, CI 0.70–1.45; **Fig 3D and S4 Fig**). In the trial sequential analysis, cumulative information did not reach the required information size, so there may be no statistical difference in arterial thrombotic events outcomes between the intervention group and the control group. Further trials are needed to confirm. However, the incidence of the venous thrombotic event was significantly lower rate in patients who were treated with antiplatelet therapy compared to no antiplatelet treatment (RR 0.86, 95%CI 0.76–0.98, *P* = 0.03, I^2^ = 0%; trial sequential analysis adjusted RR 0.87, CI 0.76–0.98; **Fig 3E and S5 Fig**). In the trial sequential analysis, the Z-curve crossed the conventional test boundary, but did not cross trial sequential monitoring boundaries, and the cumulative information did not reach the required information size, indicating that the traditional meta-analysis may obtain false-positive error and more trials need to be included to confirm the efficacy. No statistical difference in aggravation of illness (Progression to organ support or death among those not on invasive mechanical ventilation or ECMO) was found in studies compared antiplatelet therapy with no antiplatelet therapy (RR 0.98, 95%CI 0.93–1.03, *P* = 0.41, I^2^ = 0%) (**S7 Fig**).

### Sensitivity and subgroup analyses

Subgroup analyses showed no statistically significant incidence of all-cause death in aspirin groups (RR 0.96, 95%CI 0.89–1.02, *P* = 0.18, I^2^ = 0%) (**S8 Fig**), and there were no significant differences in the P2Y12 inhibitor group (RR 0.90, 95%CI 0.65–1.24, *P* = 0.52, I^2^ = 45%) (**S8 Fig**). Subgroup analyses showed the largely consistent incidence of survival to hospital discharge in aspirin groups (RR 1.02, 95%CI 1.00–1.04, *P* = 0.06, I^2^ = 0%) (**S8 Fig**), the *P* value is the critical value, whereas there were no significant differences in the P2Y12 inhibitor group (RR 1.01, 95%CI 0.97–1.06, *P* = 0.96, I^2^ = 55%) (**S8 Fig**). Neither result was statistically significant. Since REMAP-CAP was for critically ill patients in the full text, and detailed data from non-critically ill patients was mentioned in the supplementary file, we included data from non-critically ill patients to supplement the subgroup analysis. After including non-critically ill patients, subgroup analysis showed largely consistent results for all previously included incidence of survival to hospital discharge in the aspirin group (RR 1.02, 95%CI 1.00–1.04, *P* = 0.05, I^2^ = 0%) (**S8 Fig**), the *P* value is the critical value, whereas there was no significant differences in the P2Y12 inhibitor group (RR 1.01, 95%CI 0.93–1.08, *P* = 0.87, I^2^ = 67%) (**S8 Fig**). ACTIV-4B trial was excluded for symptomatic outpatients with COVID-19 with no available data to calculate relative risk. In our meta-analyses, neither fixed-effects nor random-effects models did we find any difference in treatment effects with and without antiplatelet therapy.

## Discussion

This systematic review and meta-analysis comprehensively assess the available evidence of randomized clinical trials (RCTs) regarding the effect of antiplatelet agents in COVID-19 patients. We originally included data from 5 randomized clinical trials, but only 4 provided available data to meet our quantitative evaluation, all of which were judged to be at low risk to some concern of bias. Only nine percent of participants completed the ACTIV-4B trial of aspirin in clinically stable outpatients with COVID-19 symptoms after enrollment because of the fewer adverse outcomes events than expected. The composite endpoint (all-cause death, venous thrombotic event or arterial thrombotic event, or hospitalization for cardiovascular or pulmonary disease) at 45 days was 0.0%, 0.7%, 1.4%, and 0.0%, respectively. The low occurrence, probably caused by the generally exhibited relatively mild conditions of outpatients, results in its information capacity being too low to conduct analysis. Therefore, ACTIV-4B could not be included in the quantitative evaluation. This study revealed that while antiplatelet drugs did not show a substantial advantage in terms of all-cause death, the upper bound of the confidence interval for all-cause death (CI 0.83–1.05) indicated that there was unlikely to be a demonstrated risk of harm associated with this treatment. A similar result was found in any thrombotic event. In addition, we also found that antiplatelet intervention was associated with a significant decrease in the venous thrombotic event but not in the arterial thrombotic event, but in the trial sequential analysis, venous thrombotic event indicated that the possibility of false-positive errors should be considered, and more trials are needed to confirm. Additionally, the ACTIV-4a trial with P2Y12 inhibition treatment in non-critically ill patients with COVID-19 demonstrated that the addition of P2Y12 inhibition to therapeutic-dose heparin did not decrease the trend of all-cause death or increase the proportion of survival to hospital discharge compared with only therapeutic anticoagulation. Such phenomenon differed from the observed results from the REMAP-CAP antiplatelet intervention trial of critically ill patients and the RECOVERY aspirin intervention trial of a mixed population of patients with mild, moderate, and severe COVID-19 symptoms. The inconsistency may be related to the fact that the study population in the ACTIV-4a was non-critically ill with a lower D-dimer level, and it showed more significant heterogeneity in figures compared with the REMAP-CAP and the RECOVERY trial. However, it is noted that major bleeding risk has been a considerable threat posed to the antiplatelet intervention group versus the non-antiplatelet group, and the results of the traditional meta-analysis showed robustness to the outcome of increased risk of major bleeding with antiplatelet therapy compared with no antiplatelet therapy in sequential analyses. We speculate that the antiplatelet therapy may reduce fatal complications in critically ill patients with COVID-19 but may increase the risk of bleeding (e.g., alveolar hemorrhage), which may not be clinically apparent. Taken together, the two opposite effects counterbalanced each other, resulting in an overall net neutral effect on the outcome of the proportion of mortality as well as the survival to hospital discharge. However, further investigation is needed to substantiate our speculation.

The pathophysiology of thromboembolism in COVID-19 is highly correlated to the inflammatory response to the virus, endothelial dysfunction, abnormalities of blood flow, and hypercoagulability [25, 30]. It is believed that the excessive inflammatory response caused by COVID-19 plays a vital role in the pathogenesis of thrombosis (thrombo-inflammation), including pulmonary microthrombosis and pulmonary intravascular coagulopathy [31]. Findings from previous studies and reviews have suggested that patients with COVID-19 have high rates of venous thromboembolism (VTE) and disseminated intravascular coagulation (DIC) [3, 32]. Thrombosis is the leading cause of clinical deterioration and death in COVID-19 cases, and worldwide attention is focused on whether improved or long-term anticoagulation therapy can improve clinical outcomes [25]. The initial strategy of heparin anticoagulation dosing did not result in longer survival to hospital discharge or longer duration of free cardiovascular or respiratory support in critically ill patients with COVID-19 compared with thromboprophylaxis heparin treatment [33–35]. However, an opposite result was observed in non-critically ill patients [36]. Besides, in high-risk patients hospitalized or discharged with COVID-19, thromboprophylaxis with rivaroxaban has exhibited improved clinical outcomes compared with the absence of extended thromboprophylaxis, mainly by decreasing fatal or asymptomatic thromboembolism and cardiovascular death [37]. All these trials have confirmed anticoagulation as a primary treatment for COVID-19 patients.

Platelets in COVID-19 patients are activated and highly aggregated. Activated platelets can mutually upregulate systemic inflammation, so inhibition of platelets may have antithrombotic and anti-inflammatory effects [38–41]. Although antiplatelet agents have systemic antithrombotic effects, several meta-analyses based on observational data demonstrated that while aspirin was associated with benefits regarding mortality [42, 43], the general case for antiplatelet agents is not in accordance [44, 45]. This finding may be related to the antiviral and anti-inflammatory effects of aspirin that other antiplatelet drugs do not have [46]. To further determine the previous conclusions, we plan to perform a subgroup analysis based on the severity of included patients with COVID-19 and the variety of antiplatelet agents allocated to patients in the intervention group. Due to limited data available in recently published RCTs, only a subgroup analysis of the variety of antiplatelet agents was conducted. The result suggested no significant difference in the all-cause death outcome between the aspirin subgroup and the P2Y12 inhibitors subgroup. Although no statistically significant result for survival to hospital discharge in both groups was observed, the results may have some guiding value for clinical practice. Additionally, both subgroups showed an equally evident risk of major bleeding. Our results from available data failed to show consistency with previous evidence from observational studies on all-cause death benefits from aspirin. The sequential analysis of antiplatelet intervention showed that the available data were insufficient to reach a concrete conclusion about the result of all-cause death but major bleeding. More randomized clinical trials based on different categories of antiplatelet agents are imperative for future research and a more rigorous conclusion.

### Strengths and limitations

This systematic review and meta-analysis have several strengths. First, this article comprehensively assesses the available evidence from randomized clinical trials regarding the effect of antiplatelet agents in individuals with COVID-19. We performed a trial sequential analysis to test the strength of the results (Trial sequential analysis (TSA) is similar to the interim analysis in clinical trials). The study fills a void in current academic research on the effect of antiplatelet agents, which is largely based on observational studies. Furthermore, our conclusion is highly generalizable due to the diverse geographic origins of the patients in these studies and the severity of their illness, ranging from outpatients to hospitalized patients, including non-critically ill and critically ill. Moreover, intervention including various antiplatelet agents from aspirin to different types of P2Y12 inhibitors was based on different doses or categories of anticoagulation agents, which may span the majority of patient types with COVID-19. Notably, all studies included in this meta-analysis had a high quality with a low risk of some concern of bias.

Several limitations apply to this systematic review and meta-analysis as well. First, only five RCTs were included in this article, with follow-up time for all-cause death ranging from 21 days to 90 days. This limited our possibilities to synthesize the data optimally and may cause heterogeneity. Hence, we hope to obtain more evidence regarding the outcomes of antiplatelet agents in a long-term follow-up in future studies. Second, subgroup analysis of the severity of patients was insufficient since specific data of primary and secondary outcomes based on non-critically and critically ill patients were not offered in the RECOVERY trial, which was based on a mixed population of non-critically ill and critically ill patients. Third, the obtained results do not support the routine empirical use of antiplatelet agents in non-critically ill patients due to the limited evidence from RCTs and the inconsistency observed in the ACTIV-4a trial compared with the REMAP-CAP and the RECOVERY trial, which needs to be further confirmed. Finally, given the time frame of all included trials, patients infected with COVID-19 variants, such as the omicron or delta variant may be underrepresented in the included participants’ sample. Similarly, participants who were vaccinated or treated with monoclonal antibodies before to randomization seemed to be few at the very beginning. Therefore, more timely research is in urgent need to inform new initiatives. It is worth noting that more intensive research is underway to investigate the different types of antiplatelet agents or different D-dimer levels at baseline, such as NCT04324463, NCT04363840, NCT04410328, NCT04391179, NCT04445623, NCT04365309, NCT02735707, which could provide a more comprehensive understanding of the effect of antiplatelet agents in the near future.

## Conclusion

This systematic review and meta-analysis examined data gathered from 5 recently published RCTs. The outcome revealed that while the use of antiplatelet agents exhibited no significant benefit on all-cause death, the upper bound of the confidence interval on all-cause death (CI 0.83–1.05) suggested that it was unlikely to be a substantiated harm risk associated with this treatment. Moreover, a slight benefit of all-cause death and survival to hospital discharge was observed in critically ill patients but not in non-critically ill cases, which needs further confirmation. However, evidence from all RCTs suggested a high risk of major bleeding in antiplatelet agent treatments. According to the results of our sequential analysis, the evidence available to support or negate the use of antiplatelet agents in COVID-19 patients is lacking. The results of ongoing and future well-designed, large, randomized clinical trials are needed.

## Supporting information

S1 Checklist PRISMA 2020 checklist. (DOCX)

S1 Fig Trial sequential analysis of all-cause death. https://figshare.com/ndownloader/files/42480849.
(TIF)

S2 Fig Trial sequential analysis of survival to hospital discharge. https://figshare.com/ndownloader/files/42481368.
(TIF)

S3 Fig Trial sequential analysis of any thrombotic events. https://figshare.com/ndownloader/files/42481401.
(TIF)

S4 Fig Trial sequential analysis of arterial thrombotic events. https://figshare.com/ndownloader/files/42481416.
(TIF)

S5 Fig Trial sequential analysis of venous thrombotic events. https://figshare.com/ndownloader/files/42481452.
(TIF)

S6 Fig Trial sequential analysis of major bleeding. https://figshare.com/ndownloader/files/42481476.
(TIF)

S7 Fig Forest plot depicting the pooled risk ratio of aggravation of illness between antiplatelet therapy and no antiplatelet therapy. https://figshare.com/ndownloader/files/42481593.
(TIF)

S8 Fig A. Forest plot depicting the pooled risk ratio of all-cause death for aspirin group between antiplatelet therapy and no antiplatelet therapy; B. Forest plot depicting the pooled risk ratio of all-cause death for P2Y12 group between antiplatelet therapy and no antiplatelet therapy; C. Forest plot depicting the pooled risk ratio of survival to hospital discharge for aspirin group between antiplatelet therapy and no antiplatelet therapy; D. Forest plot depicting the pooled risk ratio of survival to hospital discharge for P2Y12 inhibitor group between antiplatelet therapy and no antiplatelet therapy; E. Forest plot depicting the pooled risk ratio of survival to hospital discharge (including non-critically ill patients) for aspirin group between antiplatelet therapy and no antiplatelet therapy; F. Forest plot depicting the pooled risk ratio of survival to hospital discharge (including non-critically ill patients) for P2Y12 inhibitor group between antiplatelet therapy and no antiplatelet therapy. https://figshare.com/ndownloader/files/42481599.
(TIF)

S1 Table PRISMA 2020 checklist. https://figshare.com/ndownloader/files/42480492.
(DOCX)

S2 Table Search strategies. https://figshare.com/ndownloader/files/42480543.
(DOCX)

S3 Table PICOs criteria for inclusion and exclusion of studies. https://figshare.com/ndownloader/files/42480687.
(DOCX)

S4 Table Patient baseline characteristics. https://figshare.com/ndownloader/files/42480702.
(DOCX)

S5 Table Description of primary outcome and secondary outcomes of meta-analysis. https://figshare.com/ndownloader/files/42480723.
(DOCX)

S6 Table Description of primary outcome and secondary outcomes of meta-analysis for aspirin vs. P2Y12 inhibitor. https://figshare.com/ndownloader/files/42480768.
(DOCX)

S7 Table Comprehensive overview of included trials. https://figshare.com/ndownloader/files/42480801.
(DOCX)

S8 Table Quality assessment of included studies by risk of bias 2.0. https://figshare.com/ndownloader/files/42480810.
(DOCX)

S9 Table Evidence table for outcome measure. https://figshare.com/ndownloader/files/42480819.
(DOCX)
