# Supplementary figures and images for: Effect of antiplatelet therapy after COVID-19 diagnosis: A systematic review with meta-analysis and trial sequential analysis
Source: PLoS One. 2024 Feb 1;19(2):e0297628. doi: 10.1371/journal.pone.0297628 (PMC10833506; doi:10.1371/journal.pone.0297628)

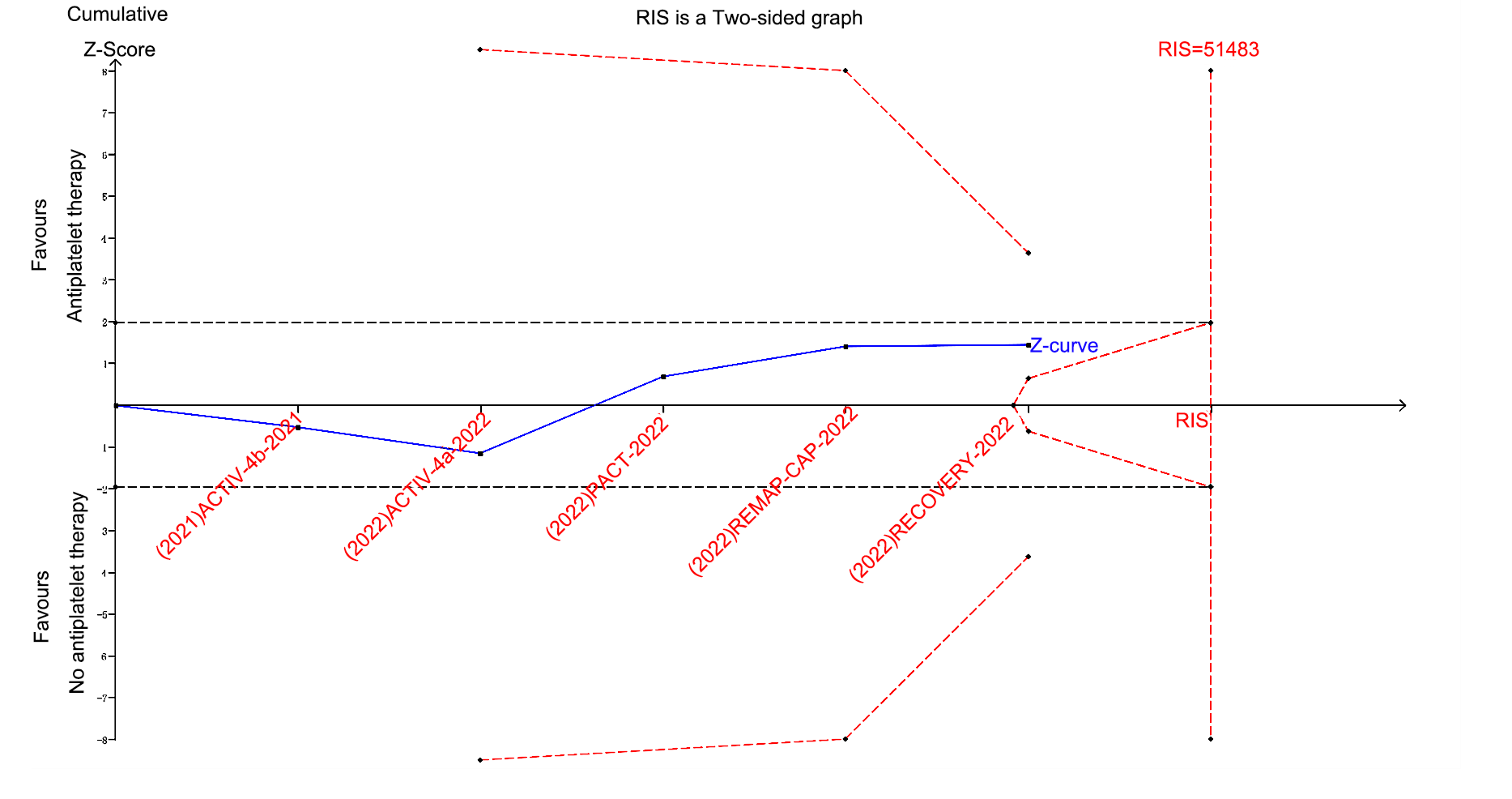

Supplement: S1 Fig — https://figshare.com/ndownloader/files/42480849. (TIF) [file pone.0297628.s002.tif]

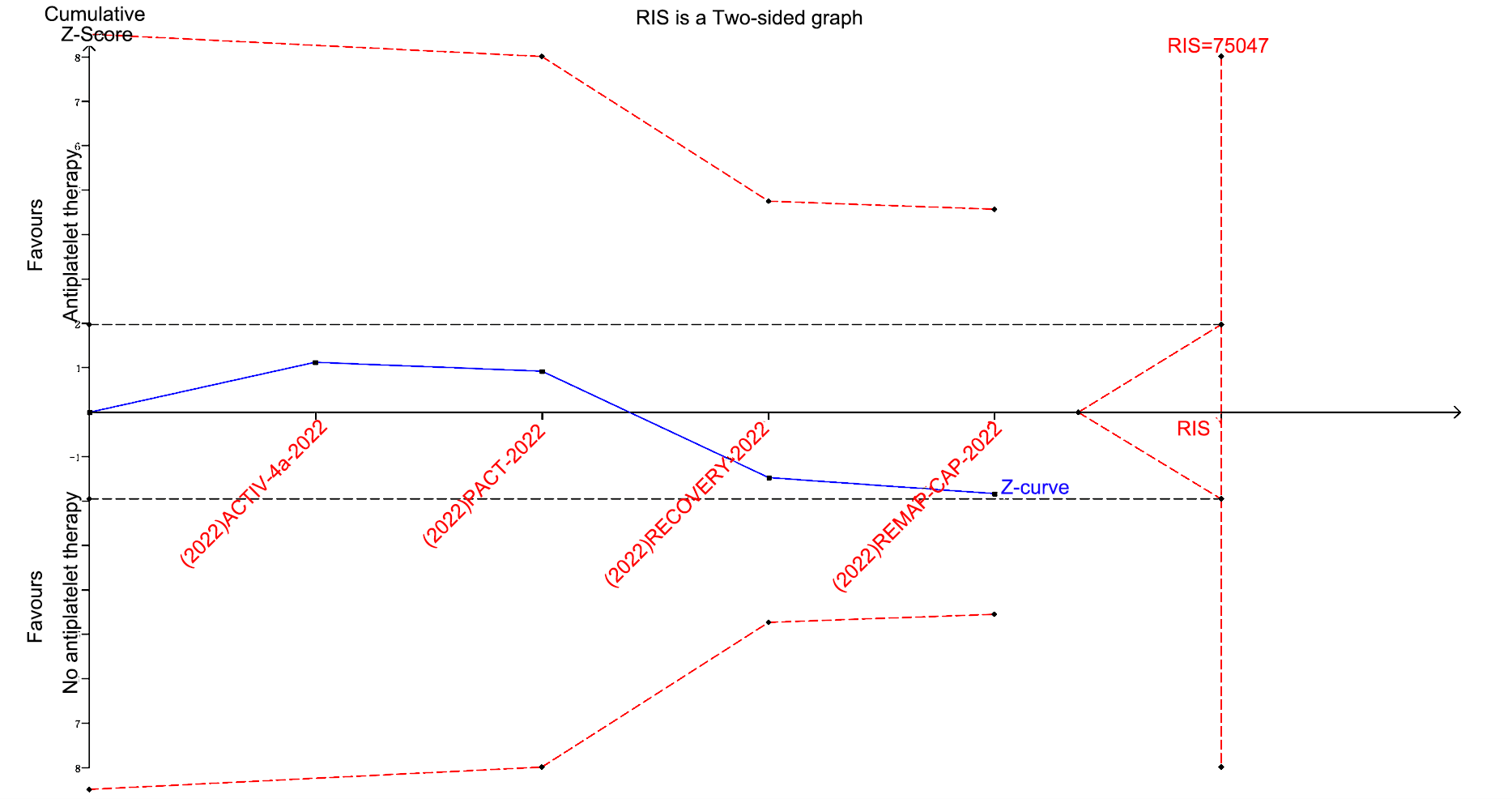

Supplement: S2 Fig — https://figshare.com/ndownloader/files/42481368. (TIF) [file pone.0297628.s003.tif]

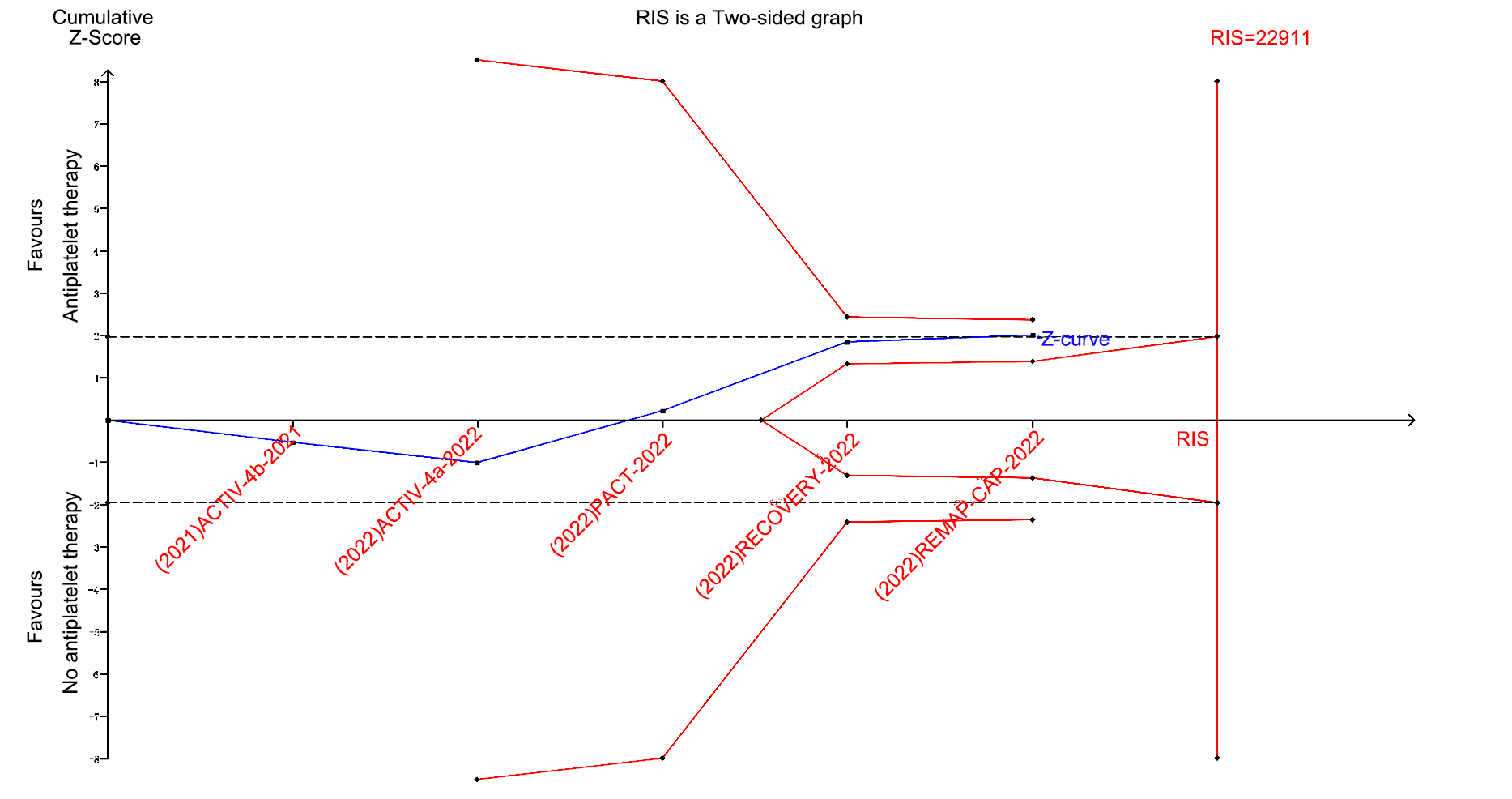

Supplement: S3 Fig — https://figshare.com/ndownloader/files/42481401. (TIF) [file pone.0297628.s004.tif]

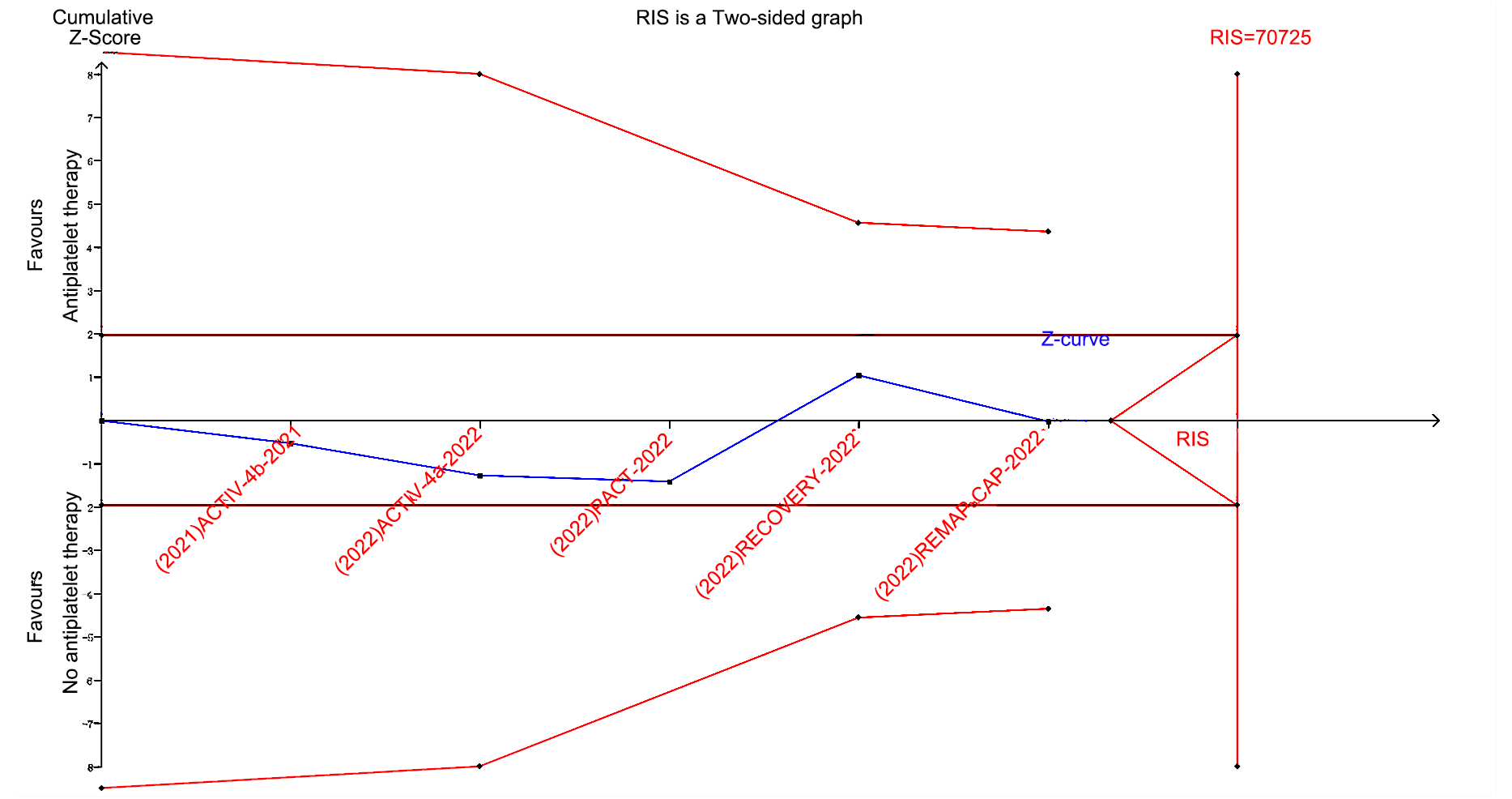

Supplement: S4 Fig — https://figshare.com/ndownloader/files/42481416. (TIF) [file pone.0297628.s005.tif]

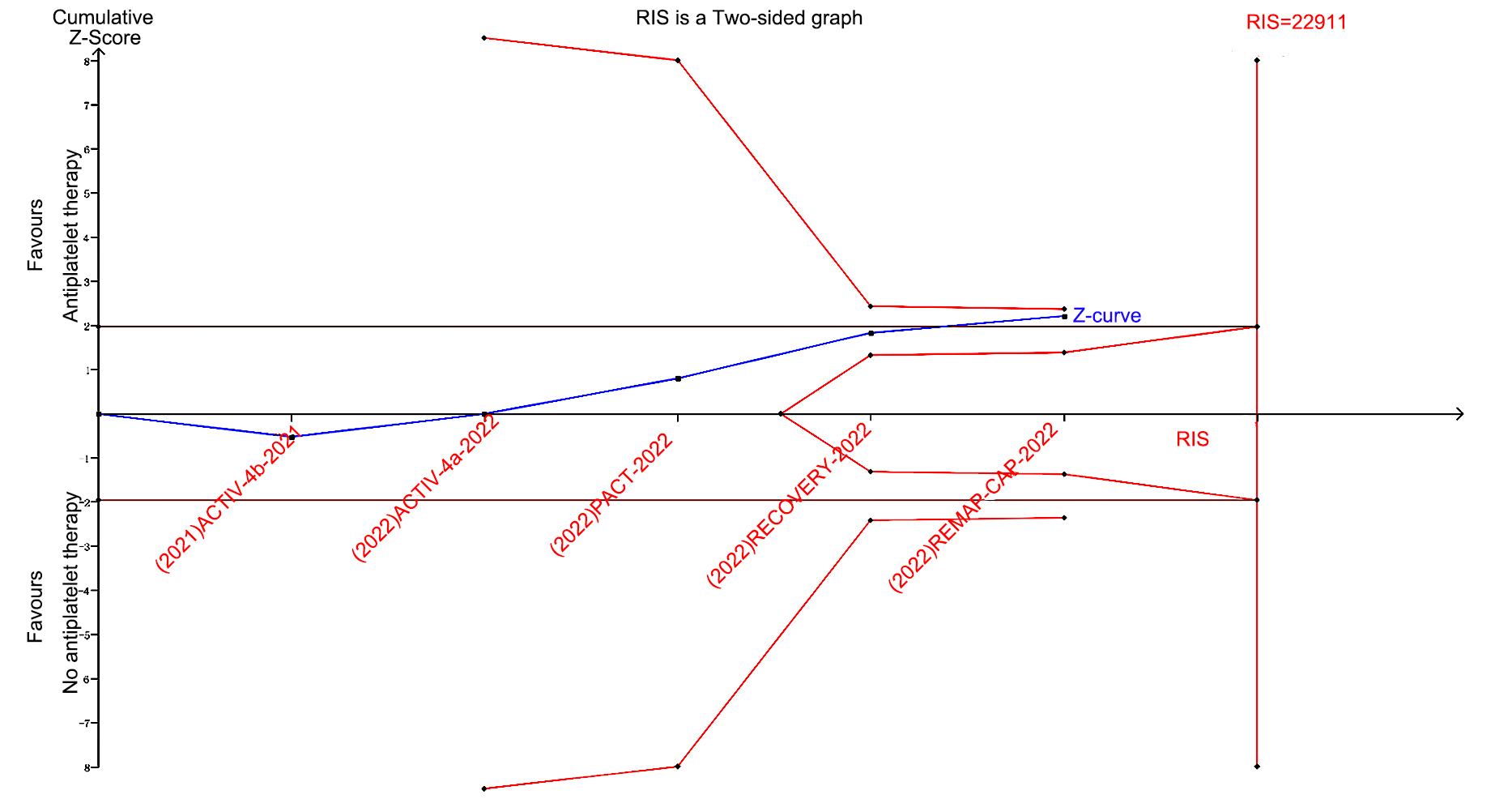

Supplement: S5 Fig — https://figshare.com/ndownloader/files/42481452. (TIF) [file pone.0297628.s006.tif]

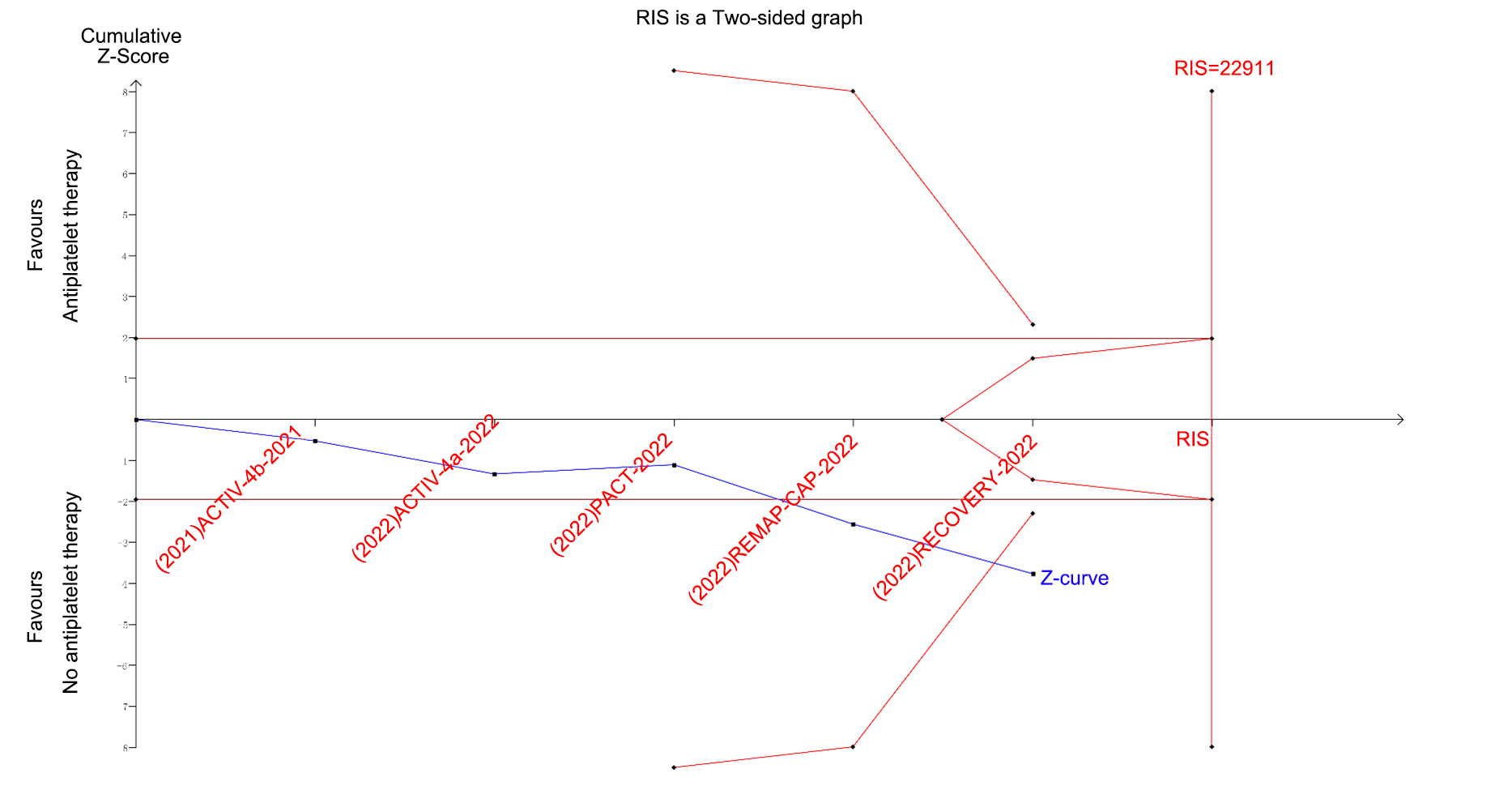

Supplement: S6 Fig — https://figshare.com/ndownloader/files/42481476. (TIF) [file pone.0297628.s007.tif]

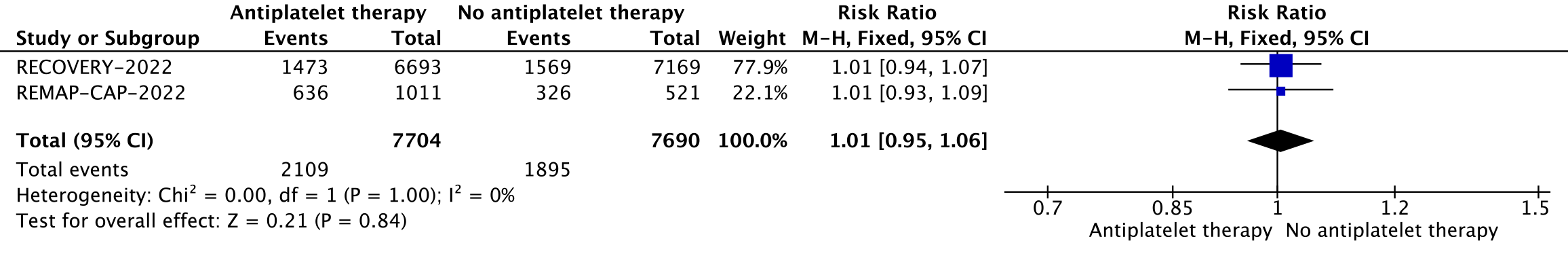

Supplement: S7 Fig — https://figshare.com/ndownloader/files/42481593. (TIF) [file pone.0297628.s008.tif]

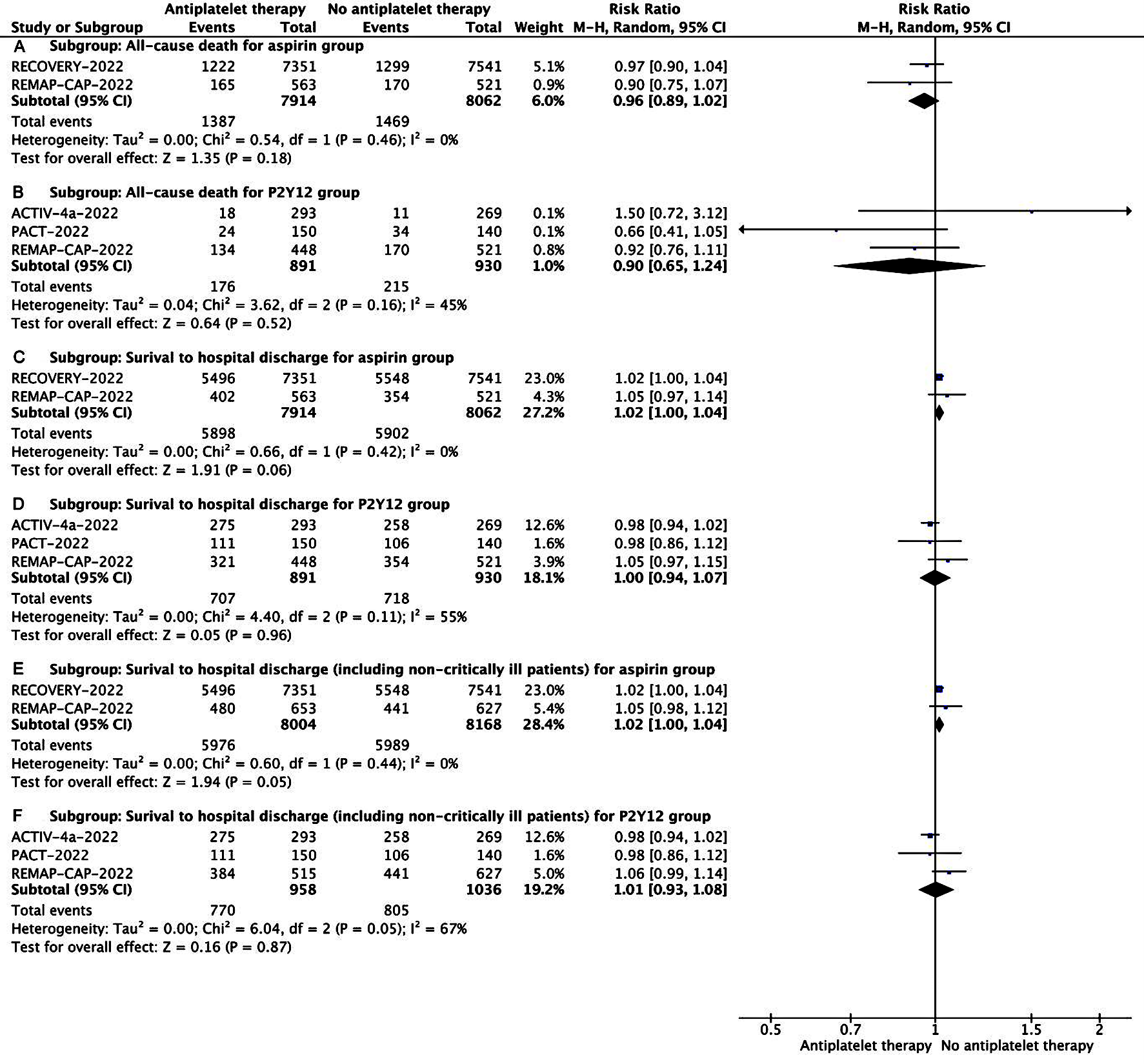

Supplement: S8 Fig — A. Forest plot depicting the pooled risk ratio of all-cause death for aspirin group between antiplatelet therapy and no antiplatelet therapy; B. Forest plot depicting the pooled risk ratio of all-cause death for P2Y12 group between antiplatelet therapy and no antiplatelet therapy; C. Forest plot depicting the pooled risk ratio of survival to hospital discharge for aspirin group between antiplatelet therapy and no antiplatelet therapy; D. Forest plot depicting the pooled risk ratio of survival to hospital discharge for P2Y12 inhibitor group between antiplatelet therapy and no antiplatelet therapy; E. Forest plot depicting the pooled risk ratio of survival to hospital discharge (including non-critically ill patients) for aspirin group between antiplatelet therapy and no antiplatelet therapy; F. Forest plot depicting the pooled risk ratio of survival to hospital discharge (including non-critically ill patients) for P2Y12 inhibitor group between antiplatelet therapy and no antiplatelet therapy. https://figshare.com/ndownloader/files/42481599. (TIF) [file pone.0297628.s009.tif]
