# Supplementary material for: Effect of antiplatelet therapy after COVID-19 diagnosis: A systematic review with meta-analysis and trial sequential analysis
Source: PLoS One. 2024 Feb 1;19(2):e0297628. doi: 10.1371/journal.pone.0297628 (PMC10833506; doi:10.1371/journal.pone.0297628)
Supplement: S2 Table — https://figshare.com/ndownloader/files/42480543. (DOCX) [file pone.0297628.s011.docx]

**Table S2: Search strategies**

**PubMed**

((((((((((((((((((((((((((((((((((((Aggregation Inhibitors, Platelet) OR (Inhibitors, Platelet Aggregation)) OR (Blood Platelet Antiaggregants)) OR (Antiaggregants, Blood Platelet)) OR (Antiaggregants, Platelet)) OR (Platelet Antiaggregant)) OR (Antiaggregant, Platelet)) OR (Blood Platelet Aggregation Inhibitor)) OR (Blood Platelet Antiaggregant)) OR (Antiaggregant, Blood Platelet)) OR (Platelet Antiaggregant, Blood)) OR (Platelet Aggregation Inhibitor)) OR (Aggregation Inhibitor, Platelet)) OR (Inhibitor, Platelet Aggregation)) OR (Blood Platelet Aggregation Inhibitors)) OR (Platelet Antiaggregants)) OR (Platelet Inhibitors)) OR (Inhibitors, Platelet)) OR (Platelet Inhibitor)) OR (Inhibitor, Platelet)) OR (Antiplatelet Agents)) OR (Agents, Antiplatelet)) OR (Antiplatelet Agent)) OR (Agent, Antiplatelet)) OR (Antiplatelet Drug)) OR (Drug, Antiplatelet)) OR (Antiplatelet Drugs)) OR (Drugs, Antiplatelet)) OR (Platelet Antagonists)) OR (Antagonists, Platelet)) OR (Blood Platelet Antagonist)) OR (Antagonist, Blood Platelet)) OR (Platelet Antagonist, Blood)) OR (Platelet Antagonist)) OR (Antagonist, Platelet)) OR (Blood Platelet Antagonists)) OR (Antagonists, Blood Platelet)

((((((((((((((((((Aspirin) OR (Acetylsalicylic Acid)) OR (Acid, Acetylsalicylic)) OR (2-(Acetyloxy)benzoic Acid)) OR (Acylpyrin)) OR (Aloxiprimum)) OR (Colfarit)) OR (Dispril)) OR (Easprin)) OR (Ecotrin)) OR (Endosprin)) OR (Magnecyl)) OR (Micristin)) OR (Polopirin)) OR (Polopiryna)) OR (Solprin)) OR (Solupsan)) OR (Zorprin)) OR (Acetysal)

((((((ticagrelor) OR (Brilique)) OR (AZD 6140)) OR (AZD6140)) OR (AZD-6140)) OR(Brilinta))OR(3-(7-((2-(3,4-Difluorophenyl)cyclopropyl)amino)-5-(propylthio)-3H-(1-3)-triazolo(4,5-d)pyrimidin-3-yl)-5-(2-hydroxyethoxy)cyclopentane-1,2-diol)

((((((((((((((((Clopidogrel) OR (SC 25989C)) OR (SC 25990C)) OR (SR 25989)) OR (Clopidogrel-Mepha)) OR (Clopidogrel Mepha)) OR (Clopidogrel Sandoz)) OR (Iscover)) OR (Clopidogrel Napadisilate)) OR (Clopidogrel Hydrochloride)) OR (PCR 4099)) OR (PCR-4099)) OR (Clopidogrel Besylate)) OR (Clopidogrel Besilate)) OR (Clopidogrel, (+)(S)-isomer)) OR (Plavix)) OR (Clopidogrel Bisulfate)

(((((((((((((((((((((((((((((((((((COVID 19) OR (SARS-CoV-2 Infection)) OR (Infection, SARS-CoV-2)) OR (SARS CoV 2 Infection)) OR (SARS-CoV-2 Infections)) OR (2019 Novel Coronavirus Disease)) OR (2019 Novel Coronavirus Infection)) OR (2019-nCoV Disease)) OR (2019 nCoV Disease)) OR (2019-nCoV Diseases)) OR (Disease, 2019-nCoV)) OR (COVID-19 Virus Infection)) OR (COVID 19 Virus Infection)) OR (COVID-19 Virus Infections)) OR (Infection, COVID-19 Virus)) OR (Virus Infection, COVID-19)) OR (Coronavirus Disease 2019)) OR (Disease 2019, Coronavirus)) OR (Coronavirus Disease-19)) OR (Coronavirus Disease 19)) OR (Severe Acute Respiratory Syndrome Coronavirus 2 Infection)) OR (SARS Coronavirus 2 Infection)) OR (COVID-19 Virus Disease)) OR (COVID 19 Virus Disease)) OR (COVID-19 Virus Diseases)) OR (Disease, COVID-19 Virus)) OR (Virus Disease, COVID-19)) OR (2019-nCoV Infection)) OR (2019 nCoV Infection)) OR (2019-nCoV Infections)) OR (Infection, 2019-nCoV)) OR (COVID19)) OR (COVID-19 Pandemic)) OR (COVID 19 Pandemic)) OR (Pandemic, COVID-19)) OR (COVID-19 Pandemics)

**EMBASE**

#1

'covid-19'/exp OR 'covid-19' OR 'covid 19'/exp OR 'covid 19' OR 'sars-cov-2 infection'/exp OR 'sars-cov-2 infection' OR 'infection, sars-cov-2' OR 'sars cov 2 infection'/exp OR 'sars cov 2 infection' OR 'sars-cov-2 infections' OR '2019 novel coronavirus disease'/exp OR '2019 novel coronavirus disease' OR '2019 novel coronavirus infection'/exp OR '2019 novel coronavirus infection' OR '2019-ncov disease'/exp OR '2019-ncov disease' OR '2019 ncov disease'/exp OR '2019 ncov disease' OR '2019-ncov diseases' OR 'disease, 2019-ncov' OR 'covid-19 virus infection' OR 'covid 19 virus infection' OR 'covid-19 virus infections' OR 'virus infection, covid-19' OR 'coronavirus disease 2019'/exp OR 'coronavirus disease 2019' OR 'disease 2019, coronavirus' OR 'coronavirus disease-19'/exp OR 'coronavirus disease-19' OR 'coronavirus disease 19'/exp OR 'coronavirus disease 19' OR 'severe acute respiratory syndrome coronavirus 2 infection'/exp OR 'severe acute respiratory syndrome coronavirus 2 infection' OR 'sars coronavirus 2 infection'/exp OR 'sars coronavirus 2 infection' OR 'covid-19 virus disease' OR 'covid 19 virus disease' OR 'disease, covid-19 virus' OR 'virus disease, covid-19' OR '2019-ncov infection'/exp OR '2019-ncov infection' OR '2019 ncov infection'/exp OR '2019 ncov infection' OR '2019-ncov infections' OR 'infection, 2019-ncov' OR 'covid19'/exp OR 'covid19' OR 'covid-19 pandemic' OR 'covid 19 pandemic' OR 'pandemic, covid-19' OR 'covid-19 pandemics'

#2

'antiplatelet' OR 'antiplatelet effect' OR 'antithrombocytic activity' OR 'antithrombocytic effect'

#3

'aspirin'/exp OR 'acetylsalicylic acid' OR '2 acetoxybenzoate' OR '2 acetoxybenzoic acid' OR '8-hour bayer' OR 'acenterine' OR 'acesal' OR 'acetan' OR 'acetard' OR 'aceticil' OR 'aceticyl' OR 'acetilum' OR 'acetonyl' OR 'acetophen' OR 'acetosal' OR 'acetosalicylic acid' OR 'acetosalin' OR 'acetosalum' OR 'acetyl salicylate' OR 'acetyl salicylic acid' OR 'acetylic salicylic acid' OR 'acetylin' OR 'acetylo' OR 'acetylo salicylic acid' OR 'acetylon' OR 'acetylosalicylic acid' OR 'acetylsal' OR 'acetylsalicyclic acid' OR 'acetylsalicyl' OR 'acetylsalicylate' OR 'acetylsalicylate strontium' OR 'acetylsalicylic acid plus glycine' OR 'acetylsalicylic acid sodium salt' OR 'acetylsalicylic acid strontium salt' OR 'acetylsalycic acid' OR 'acetylsalycylic acid' OR 'acetysal' OR 'acidulatum' OR 'acidum acetyl salicylicum' OR 'acidum acetylosalicylicum' OR 'acidum acetylsalicylicum' OR 'actorin' OR 'acylpyrin' OR 'acylpyrine' OR 'acytosal' OR 'adiro' OR 'alabukun' OR 'alasil' OR 'albyl e' OR 'albyl minor' OR 'albyl-e' OR 'alka seltzer' OR 'alka-seltzer' OR 'alkaspirin' OR 'anasprin' OR 'andol' OR 'anopyrin' OR 'ansin' OR 'anthrom' OR 'aptor' OR 'arthralgyl' OR 'arthritis strength bufferin' OR 'asa' OR 'asa akut' OR 'asa cardio' OR 'asa direk' OR 'asa effect' OR 'asa express' OR 'asa migraene' OR 'asa migrane' OR 'asa migren' OR 'asa pro' OR 'asa protect' OR 'asa ultra' OR 'asa ultra fast' OR 'asa zipp' OR 'asaa' OR 'asaa gr' OR 'asaa microactive' OR 'asaa rapida' OR 'asacard' OR 'asae' OR 'asae bruis' OR 'asae ec protect' OR 'asae fasttabs' OR 'asae protect' OR 'asaetta' OR 'asaflow' OR 'asaphen' OR 'asaphen e.c.' OR 'asapor' OR 'asatard' OR 'asawin' OR 'aspec' OR 'aspec-ec' OR 'aspent' OR 'aspergum' OR 'aspex' OR 'aspilets' OR 'aspirem' OR 'aspirgran' OR 'aspiricor' OR 'aspirin' OR 'aspirin bayer' OR 'aspirina' OR 'aspirine' OR 'aspirinine' OR 'aspirisucre' OR 'aspisol' OR 'aspo cid' OR 'aspro' OR 'aspro cardio' OR 'aspro clear' OR 'asproflash' OR 'asrina' OR 'asrivo' OR 'asta' OR 'asteric' OR 'asteric acid' OR 'astrix' OR 'bamyl' OR 'bayaspirina' OR 'bayer aspirin' OR 'bayer aspirin cardio' OR 'bayer extra strength aspirin for migraine pain' OR 'bebesan' OR 'biprin' OR 'bokey' OR 'boxazin' OR 'breoprin' OR 'buffered aspirin' OR 'bufferin' OR 'bufferin low dose' OR 'cafenol' OR 'caprin (acetylsalicylic acid)' OR 'caprin (aspirin)' OR 'cardioasa' OR 'cardioasae' OR 'cardioaspirina' OR 'cardioflow (acetylsalicylic acid)' OR 'cartia' OR 'caspirin' OR 'catalgine' OR 'catalgix' OR 'cemerit' OR 'cemirit' OR 'claradin' OR 'claragine' OR 'colfarit' OR 'comoprin' OR 'contrheuma' OR 'contrheuma retard' OR 'darosal' OR 'depot aspirin' OR 'dispirin' OR 'dolean' OR 'durlaza' OR 'dusil' OR 'easprin' OR 'ecasil' OR 'ecosprin' OR 'ecotrin' OR 'ecotrin 650' OR 'egalgic' OR 'emocin' OR 'empirin' OR 'encaprin' OR 'encine em' OR 'endosprin' OR 'entaprin' OR 'entericin' OR 'enteroprin' OR 'enterosarine' OR 'enterospirine' OR 'entrophen' OR 'eskotrin' OR 'euthermine' OR 'extren' OR 'flamasacard' OR 'genasprin' OR 'globentyl' OR 'godamed' OR 'gotosan' OR 'helicon' OR 'herz ass' OR 'hjertemagnyl' OR 'idotyl' OR 'infatabs a' OR 'istopirin' OR 'istopyrine' OR 'ivepirine' OR 'juvepirine' OR 'keypo' OR 'kilios' OR 'kinderaspirin' OR 'magnecyl brus' OR 'magnyl dak' OR 'mcn r 358' OR 'measurin' OR 'mejoral' OR 'melabon' OR 'micristin' OR 'micropyrin' OR 'migrasaa' OR 'mikristin' OR 'miniasal' OR 'mycristin' OR 'naspro' OR 'novasen' OR 'nu seal' OR 'nu-seals' OR 'nu-seals asa' OR 'nuseals' OR 'ortho acetoxybenzoate' OR 'ortho acetoxybenzoic acid' OR 'ortho acetyloxybenzoate' OR 'ortho acetyloxybenzoic acid' OR 'ostoprin' OR 'pancemol' OR 'para acetylsalicylic acid' OR 'paracin' OR 'paynocil' OR 'pengo' OR 'platet 300 cleartab' OR 'plewin' OR 'polopiryna' OR 'premaspin' OR 'primaspan' OR 'proprin' OR 'pyronoval' OR 'reumyl' OR 'rhodine' OR 'rhonal' OR 'rhonal for children' OR 'ronal' OR 'salacetin' OR 'salacetogen' OR 'saletin' OR 'salisalido' OR 'salospir' OR 'sargepirine' OR 'sedergine' OR 'sedergine forte' OR 'slow release aspirin' OR 'sodium acetylsalicylate' OR 'sodium bicarbonate acetyl salicylate' OR 'sodium bicarbonate acetylsalicylate' OR 'soldral' OR 'solpyron' OR 'solucetyl' OR 'solupsa' OR 'spren' OR 'super tru' OR 'tapal' OR 'temagin' OR 'tevapirin' OR 'th 2152' OR 'thrombo-aspilets' OR 'toldex retard' OR 'treupahlin' OR 'treuphalin' OR 'tromalyt' OR 'tromcor' OR 'turivital' OR 'vazalore' OR 'verin' OR 'vitalink' OR 'xaxa' OR 'ysp aspirin' OR 'zero-order release' OR 'zorprin'

#4

'ticagrelor'/exp OR '3 [7 [2 (3, 4 difluorophenyl) cyclopropylamino] 5 propylthio 1, 2, 3 triazolo [4, 5 d] pyrimidin 3 yl] 5 (2 hydroxyethoxy) 1, 2 cyclopentanediol' OR '3 [7 [2 (3, 4 difluorophenyl) cyclopropylamino] 5 propylthio 1, 2, 3 triazolo [4, 5 d] pyrimidin 3 yl] 5 hydroxymethoxy 1, 2 cyclopentanediol' OR '3 [7 [ [2 (3, 4 difluorophenyl) cyclopropyl] amino] 5 (propylsulfanyl) 3h 1, 2, 3 triazolo [4, 5 d] pyrimidin 3 yl] 5 (2 hydroxyethoxy) cyclopentane 1, 2 diol' OR 'ar c126532' OR 'arc126532' OR 'azd 6140' OR 'azd6140' OR 'brilinta' OR 'brilique' OR 'possia'

#5

'clopidogrel'/exp OR '2 (2 chlorophenyl) 2 (4, 5, 6, 7 tetrahydrothieno [3, 2 c] pyridin 5 yl) acetic acid methyl ester' OR 'alpha (2 chlorophenyl) 6, 7 dihydrothieno [3, 2 c] pyridine 5 (4h) acetic acid methyl ester' OR 'clopidogrel besilate' OR 'clopidogrel besylate' OR 'clopidogrel bisulfate' OR 'clopidogrel bisulphate' OR 'clopidogrel bms' OR 'clopidogrel hcs' OR 'clopidogrel hydrobromide' OR 'clopidogrel hydrochloride' OR 'clopidogrel hydrogen sulfate' OR 'clopidogrel hydrogen sulphate' OR 'clopidogrel winthrop' OR 'clopilet' OR 'grepid' OR 'inhiplat' OR 'iscover' OR 'mdco 157' OR 'mdco157 or methyl 2 (2 chlorophenyl) 2 (6, 7 dihydro 4h thieno [3, 2 c] pyridin 5 yl) acetate' OR 'myogrel' OR 'osvix' OR 'pcr 4099' OR 'pcr4099' OR 'plavitor' OR 'plavix' OR 'pm 103' OR 'pm103' OR 'pregrel' OR 'sr 25989' OR 'sr 25990c' OR 'sr25989' OR 'sr25990c' OR 'thrombo (drug)' OR 'zopya' OR 'zylagren' OR 'zyllt'

#6

#2 OR #3 OR #4 OR #5

#7

#1 AND #6

**Web of Science**

TS=(((((((((((((((((((((((((((((((((((((Aggregation Inhibitors, Platelet) OR (Inhibitors, Platelet Aggregation)) OR (Blood Platelet Antiaggregants)) OR (Antiaggregants, Blood Platelet)) OR (Antiaggregants, Platelet)) OR (Platelet Antiaggregant)) OR (Antiaggregant, Platelet)) OR (Blood Platelet Aggregation Inhibitor)) OR (Blood Platelet Antiaggregant)) OR (Antiaggregant, Blood Platelet)) OR (Platelet Antiaggregant, Blood)) OR (Platelet Aggregation Inhibitor)) OR (Aggregation Inhibitor, Platelet)) OR (Inhibitor, Platelet Aggregation)) OR (Blood Platelet Aggregation Inhibitors)) OR (Platelet Antiaggregants)) OR (Platelet Inhibitors)) OR (Inhibitors, Platelet)) OR (Platelet Inhibitor)) OR (Inhibitor, Platelet)) OR (Antiplatelet Agents)) OR (Agents, Antiplatelet)) OR (Antiplatelet Agent)) OR (Agent, Antiplatelet)) OR (Antiplatelet Drug)) OR (Drug, Antiplatelet)) OR (Antiplatelet Drugs)) OR (Drugs, Antiplatelet)) OR (Platelet Antagonists)) OR (Antagonists, Platelet)) OR (Blood Platelet Antagonist)) OR (Antagonist, Blood Platelet)) OR (Platelet Antagonist, Blood)) OR (Platelet Antagonist)) OR (Antagonist, Platelet)) OR (Blood Platelet Antagonists)) OR (Antagonists, Blood Platelet))

TS=(((((((((((((((((((Aspirin) OR (Acetylsalicylic Acid)) OR (Acid, Acetylsalicylic)) OR (2-(Acetyloxy)benzoic Acid)) OR (Acylpyrin)) OR (Aloxiprimum)) OR (Colfarit)) OR (Dispril)) OR (Easprin)) OR (Ecotrin)) OR (Endosprin)) OR (Magnecyl)) OR (Micristin)) OR (Polopirin)) OR (Polopiryna)) OR (Solprin)) OR (Solupsan)) OR (Zorprin)) OR (Acetysal))

TS=(((((((ticagrelor) OR (Brilique)) OR (AZD 6140)) OR (AZD6140)) OR (AZD-6140)) OR (Brilinta)) OR (3-(7-((2-(3,4-Difluorophenyl)cyclopropyl)amino)-5-(propylthio)-3H-(1-3)-triazolo(4,5-d)pyrimidin-3-yl)-5-(2-hydroxyethoxy)cyclopentane-1,2-diol))

TS=(((((((((((((((((Clopidogrel) OR (SC 25989C)) OR (SC 25990C)) OR (SR 25989)) OR (Clopidogrel-Mepha)) OR (Clopidogrel Mepha)) OR (Clopidogrel Sandoz)) OR (Iscover)) OR (Clopidogrel Napadisilate)) OR (Clopidogrel Hydrochloride)) OR (PCR 4099)) OR (PCR-4099)) OR (Clopidogrel Besylate)) OR (Clopidogrel Besilate)) OR (Clopidogrel, (+)(S)-isomer)) OR (Plavix)) OR (Clopidogrel Bisulfate))

#4 OR #3 OR #2 OR #1

TS=((((((((((((((((((((((((((((((((((((COVID 19) OR (SARS-CoV-2 Infection)) OR (Infection, SARS-CoV-2)) OR (SARS CoV 2 Infection)) OR (SARS-CoV-2 Infections)) OR (2019 Novel Coronavirus Disease)) OR (2019 Novel Coronavirus Infection)) OR (2019-nCoV Disease)) OR (2019 nCoV Disease)) OR (2019-nCoV Diseases)) OR (Disease, 2019-nCoV)) OR (COVID-19 Virus Infection)) OR (COVID 19 Virus Infection)) OR (COVID-19 Virus Infections)) OR (Infection, COVID-19 Virus)) OR (Virus Infection, COVID-19)) OR (Coronavirus Disease 2019)) OR (Disease 2019, Coronavirus)) OR (Coronavirus Disease-19)) OR (Coronavirus Disease 19)) OR (Severe Acute Respiratory Syndrome Coronavirus 2 Infection)) OR (SARS Coronavirus 2 Infection)) OR (COVID-19 Virus Disease)) OR (COVID 19 Virus Disease)) OR (COVID-19 Virus Diseases)) OR (Disease, COVID-19 Virus)) OR (Virus Disease, COVID-19)) OR (2019-nCoV Infection)) OR (2019 nCoV Infection)) OR (2019-nCoV Infections)) OR (Infection, 2019-nCoV)) OR (COVID19)) OR (COVID-19 Pandemic)) OR (COVID 19 Pandemic)) OR (Pandemic, COVID-19)) OR (COVID-19 Pandemics))

#6 AND #5

**Cochrane**

#1

'covid-19'/exp OR 'covid-19' OR 'covid 19'/exp OR 'covid 19' OR 'sars-cov-2 infection'/exp OR 'sars-cov-2 infection' OR 'infection, sars-cov-2' OR 'sars cov 2 infection'/exp OR 'sars cov 2 infection' OR 'sars-cov-2 infections' OR '2019 novel coronavirus disease'/exp OR '2019 novel coronavirus disease' OR '2019 novel coronavirus infection'/exp OR '2019 novel coronavirus infection' OR '2019-ncov disease'/exp OR '2019-ncov disease' OR '2019 ncov disease'/exp OR '2019 ncov disease' OR '2019-ncov diseases' OR 'disease, 2019-ncov' OR 'covid-19 virus infection' OR 'covid 19 virus infection' OR 'covid-19 virus infections' OR 'virus infection, covid-19' OR 'coronavirus disease 2019'/exp OR 'coronavirus disease 2019' OR 'disease 2019, coronavirus' OR 'coronavirus disease-19'/exp OR 'coronavirus disease-19' OR 'coronavirus disease 19'/exp OR 'coronavirus disease 19' OR 'severe acute respiratory syndrome coronavirus 2 infection'/exp OR 'severe acute respiratory syndrome coronavirus 2 infection' OR 'sars coronavirus 2 infection'/exp OR 'sars coronavirus 2 infection' OR 'covid-19 virus disease' OR 'covid 19 virus disease' OR 'disease, covid-19 virus' OR 'virus disease, covid-19' OR '2019-ncov infection'/exp OR '2019-ncov infection' OR '2019 ncov infection'/exp OR '2019 ncov infection' OR '2019-ncov infections' OR 'infection, 2019-ncov' OR 'covid19'/exp OR 'covid19' OR 'covid-19 pandemic' OR 'covid 19 pandemic' OR 'pandemic, covid-19' OR 'covid-19 pandemics'

#2

'antiplatelet' OR 'antiplatelet effect' OR 'antithrombocytic activity' OR 'antithrombocytic effect'

#3

'aspirin'/exp OR 'acetylsalicylic acid' OR '2 acetoxybenzoate' OR '2 acetoxybenzoic acid' OR '8-hour bayer' OR 'acenterine' OR 'acesal' OR 'acetan' OR 'acetard' OR 'aceticil' OR 'aceticyl' OR 'acetilum' OR 'acetonyl' OR 'acetophen' OR 'acetosal' OR 'acetosalicylic acid' OR 'acetosalin' OR 'acetosalum' OR 'acetyl salicylate' OR 'acetyl salicylic acid' OR 'acetylic salicylic acid' OR 'acetylin' OR 'acetylo' OR 'acetylo salicylic acid' OR 'acetylon' OR 'acetylosalicylic acid' OR 'acetylsal' OR 'acetylsalicyclic acid' OR 'acetylsalicyl' OR 'acetylsalicylate' OR 'acetylsalicylate strontium' OR 'acetylsalicylic acid plus glycine' OR 'acetylsalicylic acid sodium salt' OR 'acetylsalicylic acid strontium salt' OR 'acetylsalycic acid' OR 'acetylsalycylic acid' OR 'acetysal' OR 'acidulatum' OR 'acidum acetyl salicylicum' OR 'acidum acetylosalicylicum' OR 'acidum acetylsalicylicum' OR 'actorin' OR 'acylpyrin' OR 'acylpyrine' OR 'acytosal' OR 'adiro' OR 'alabukun' OR 'alasil' OR 'albyl e' OR 'albyl minor' OR 'albyl-e' OR 'alka seltzer' OR 'alka-seltzer' OR 'alkaspirin' OR 'anasprin' OR 'andol' OR 'anopyrin' OR 'ansin' OR 'anthrom' OR 'aptor' OR 'arthralgyl' OR 'arthritis strength bufferin' OR 'asa' OR 'asa akut' OR 'asa cardio' OR 'asa direk' OR 'asa effect' OR 'asa express' OR 'asa migraene' OR 'asa migrane' OR 'asa migren' OR 'asa pro' OR 'asa protect' OR 'asa ultra' OR 'asa ultra fast' OR 'asa zipp' OR 'asaa' OR 'asaa gr' OR 'asaa microactive' OR 'asaa rapida' OR 'asacard' OR 'asae' OR 'asae bruis' OR 'asae ec protect' OR 'asae fasttabs' OR 'asae protect' OR 'asaetta' OR 'asaflow' OR 'asaphen' OR 'asaphen e.c.' OR 'asapor' OR 'asatard' OR 'asawin' OR 'aspec' OR 'aspec-ec' OR 'aspent' OR 'aspergum' OR 'aspex' OR 'aspilets' OR 'aspirem' OR 'aspirgran' OR 'aspiricor' OR 'aspirin' OR 'aspirin bayer' OR 'aspirina' OR 'aspirine' OR 'aspirinine' OR 'aspirisucre' OR 'aspisol' OR 'aspo cid' OR 'aspro' OR 'aspro cardio' OR 'aspro clear' OR 'asproflash' OR 'asrina' OR 'asrivo' OR 'asta' OR 'asteric' OR 'asteric acid' OR 'astrix' OR 'bamyl' OR 'bayaspirina' OR 'bayer aspirin' OR 'bayer aspirin cardio' OR 'bayer extra strength aspirin for migraine pain' OR 'bebesan' OR 'biprin' OR 'bokey' OR 'boxazin' OR 'breoprin' OR 'buffered aspirin' OR 'bufferin' OR 'bufferin low dose' OR 'cafenol' OR 'caprin (acetylsalicylic acid)' OR 'caprin (aspirin)' OR 'cardioasa' OR 'cardioasae' OR 'cardioaspirina' OR 'cardioflow (acetylsalicylic acid)' OR 'cartia' OR 'caspirin' OR 'catalgine' OR 'catalgix' OR 'cemerit' OR 'cemirit' OR 'claradin' OR 'claragine' OR 'colfarit' OR 'comoprin' OR 'contrheuma' OR 'contrheuma retard' OR 'darosal' OR 'depot aspirin' OR 'dispirin' OR 'dolean' OR 'durlaza' OR 'dusil' OR 'easprin' OR 'ecasil' OR 'ecosprin' OR 'ecotrin' OR 'ecotrin 650' OR 'egalgic' OR 'emocin' OR 'empirin' OR 'encaprin' OR 'encine em' OR 'endosprin' OR 'entaprin' OR 'entericin' OR 'enteroprin' OR 'enterosarine' OR 'enterospirine' OR 'entrophen' OR 'eskotrin' OR 'euthermine' OR 'extren' OR 'flamasacard' OR 'genasprin' OR 'globentyl' OR 'godamed' OR 'gotosan' OR 'helicon' OR 'herz ass' OR 'hjertemagnyl' OR 'idotyl' OR 'infatabs a' OR 'istopirin' OR 'istopyrine' OR 'ivepirine' OR 'juvepirine' OR 'keypo' OR 'kilios' OR 'kinderaspirin' OR 'magnecyl brus' OR 'magnyl dak' OR 'mcn r 358' OR 'measurin' OR 'mejoral' OR 'melabon' OR 'micristin' OR 'micropyrin' OR 'migrasaa' OR 'mikristin' OR 'miniasal' OR 'mycristin' OR 'naspro' OR 'novasen' OR 'nu seal' OR 'nu-seals' OR 'nu-seals asa' OR 'nuseals' OR 'ortho acetoxybenzoate' OR 'ortho acetoxybenzoic acid' OR 'ortho acetyloxybenzoate' OR 'ortho acetyloxybenzoic acid' OR 'ostoprin' OR 'pancemol' OR 'para acetylsalicylic acid' OR 'paracin' OR 'paynocil' OR 'pengo' OR 'platet 300 cleartab' OR 'plewin' OR 'polopiryna' OR 'premaspin' OR 'primaspan' OR 'proprin' OR 'pyronoval' OR 'reumyl' OR 'rhodine' OR 'rhonal' OR 'rhonal for children' OR 'ronal' OR 'salacetin' OR 'salacetogen' OR 'saletin' OR 'salisalido' OR 'salospir' OR 'sargepirine' OR 'sedergine' OR 'sedergine forte' OR 'slow release aspirin' OR 'sodium acetylsalicylate' OR 'sodium bicarbonate acetyl salicylate' OR 'sodium bicarbonate acetylsalicylate' OR 'soldral' OR 'solpyron' OR 'solucetyl' OR 'solupsa' OR 'spren' OR 'super tru' OR 'tapal' OR 'temagin' OR 'tevapirin' OR 'th 2152' OR 'thrombo-aspilets' OR 'toldex retard' OR 'treupahlin' OR 'treuphalin' OR 'tromalyt' OR 'tromcor' OR 'turivital' OR 'vazalore' OR 'verin' OR 'vitalink' OR 'xaxa' OR 'ysp aspirin' OR 'zero-order release' OR 'zorprin'

#4

'ticagrelor'/exp OR '3 [7 [2 (3, 4 difluorophenyl) cyclopropylamino] 5 propylthio 1, 2, 3 triazolo [4, 5 d] pyrimidin 3 yl] 5 (2 hydroxyethoxy) 1, 2 cyclopentanediol' OR '3 [7 [2 (3, 4 difluorophenyl) cyclopropylamino] 5 propylthio 1, 2, 3 triazolo [4, 5 d] pyrimidin 3 yl] 5 hydroxymethoxy 1, 2 cyclopentanediol' OR '3 [7 [ [2 (3, 4 difluorophenyl) cyclopropyl] amino] 5 (propylsulfanyl) 3h 1, 2, 3 triazolo [4, 5 d] pyrimidin 3 yl] 5 (2 hydroxyethoxy) cyclopentane 1, 2 diol' OR 'ar c126532' OR 'arc126532' OR 'azd 6140' OR 'azd6140' OR 'brilinta' OR 'brilique' OR 'possia'

#5

'clopidogrel'/exp OR '2 (2 chlorophenyl) 2 (4, 5, 6, 7 tetrahydrothieno [3, 2 c] pyridin 5 yl) acetic acid methyl ester' OR 'alpha (2 chlorophenyl) 6, 7 dihydrothieno [3, 2 c] pyridine 5 (4h) acetic acid methyl ester' OR 'clopidogrel besilate' OR 'clopidogrel besylate' OR 'clopidogrel bisulfate' OR 'clopidogrel bisulphate' OR 'clopidogrel bms' OR 'clopidogrel hcs' OR 'clopidogrel hydrobromide' OR 'clopidogrel hydrochloride' OR 'clopidogrel hydrogen sulfate' OR 'clopidogrel hydrogen sulphate' OR 'clopidogrel winthrop' OR 'clopilet' OR 'grepid' OR 'inhiplat' OR 'iscover' OR 'mdco 157' OR 'mdco157 or methyl 2 (2 chlorophenyl) 2 (6, 7 dihydro 4h thieno [3, 2 c] pyridin 5 yl) acetate' OR 'myogrel' OR 'osvix' OR 'pcr 4099' OR 'pcr4099' OR 'plavitor' OR 'plavix' OR 'pm 103' OR 'pm103' OR 'pregrel' OR 'sr 25989' OR 'sr 25990c' OR 'sr25989' OR 'sr25990c' OR 'thrombo (drug)' OR 'zopya' OR 'zylagren' OR 'zyllt'

#6

#2 OR #3 OR #4 OR #5

#7

#1 AND #6
