# Supplementary material for: Effect of antiplatelet therapy after COVID-19 diagnosis: A systematic review with meta-analysis and trial sequential analysis
Source: PLoS One. 2024 Feb 1;19(2):e0297628. doi: 10.1371/journal.pone.0297628 (PMC10833506; doi:10.1371/journal.pone.0297628)
Supplement: S3 Table — https://figshare.com/ndownloader/files/42480687. (DOCX) [file pone.0297628.s012.docx]

Table S3: PICOs criteria for inclusion and exclusion of studies

|  | Inclusion criteria | Exclusion criteria |
| --- | --- | --- |
| Population | ≥18 years with COVID-19 patients | Non-COVID-19 patients, and < 18 years with COVID-19 patients |
| Intervention | Antiplatelet therapy | Other non-antiplatelet drugs such as anticoagulant drug |
| Comparison | No antiplatelet therapy | / |
| Outcome | At least one of the outcomes (mortality, major bleeding, or thromboembolism) reported | None of the outcomes (mortality, major bleeding, or thromboembolism) reported |
| Study design | Randomized control trials | Cohort studies  Observational studies  Reviews, editorials, commentaries, conference abstracts, case reports, meeting abstracts, practice guidelines， protocols. |
