# Supplementary material for: Effect of antiplatelet therapy after COVID-19 diagnosis: A systematic review with meta-analysis and trial sequential analysis
Source: PLoS One. 2024 Feb 1;19(2):e0297628. doi: 10.1371/journal.pone.0297628 (PMC10833506; doi:10.1371/journal.pone.0297628)
Supplement: S4 Table — https://figshare.com/ndownloader/files/42480702. (DOCX) [file pone.0297628.s013.docx]

Table S4: Patient Baseline Characteristics

| Study | REMAP-CAP | ACTIV-4a | RECOVERY | ACTIV-4b | PACT |
| --- | --- | --- | --- | --- | --- |
| Respiratory support received |  |  |  |  |  |
| None or simple oxygen | 246 vs. 133 | 262 vs. 238 | 4936 vs. 5036 | Not reported | 1 vs. 3 |
| Non-invasive ventilation | 400 vs. 202 | 0 vs. 0 | 2057 vs. 2133 | Not reported | 128 vs.115 |
| Invasive mechanical ventilation | 374 vs. 194 | 0 vs. 0 | 358 vs. 372 | Not reported | 21 vs. 22 |
| ECMO（extracorporeal membrane oxygenation） | 246 vs. 132 | 0 vs. 0 | Not reported | Not reported | Not reported |
| Previous diseases |  |  |  |  |  |
| Diabetes | 227 vs. 112 | 72 vs. 73 | 1588 vs. 1659 | 29 vs. 91 | 40 vs. 41 |
| Heart disease | 41 vs. 26 | Not reported | 776 vs. 788 | 55 vs. 177 | 10 vs. 9 |
| Chronic lung disease | 201 vs. 97 | 112 vs. 74 | 1425 vs. 1411 | Not reported | 31 vs. 29 |
| Liver disease | no reported | 4 vs. 8 | 67 vs. 53 | Not reported | Not reported |
| Kidney impairment | 34 vs. 18 | 11 vs. 11 | 214 vs. 251 | Not reported | 10 vs. 11 |
| Concomitant therapies |  |  |  |  |  |
| Corticosteroids | 985 vs. 511 | 192 vs. 168 | 6906 vs. 7109 | Not reported | Not reported |
| Remdesivir | 200 vs. 126 | 164 vs. 128 | 1869 vs. 1952 | Not reported | Not reported |
| Tocilizumab | 449 vs. 217 | Specific values are not reported | 946 vs. 975 | Not reported | Not reported |
| Sarilumab | 111 vs. 54 | Not reported | Specific values are not reported | Not reported | Not reported |
| Hydroxychloroquine | not reported | Not reported | 16 vs. 15 | Not reported | Not reported |
| Convalescent plasma | not reported | Not reported | 1125 vs. 1157 | Not reported | Not reported |

^A^ As for Data Processing of REMAP-CAP and ACTIV-4B: continuous variables (Race, Respiratory support received, Previous diseases, Concomitant therapies) should be represented by the post-merger mean.
