# Supplementary material for: Effect of antiplatelet therapy after COVID-19 diagnosis: A systematic review with meta-analysis and trial sequential analysis
Source: PLoS One. 2024 Feb 1;19(2):e0297628. doi: 10.1371/journal.pone.0297628 (PMC10833506; doi:10.1371/journal.pone.0297628)
Supplement: S5 Table — https://figshare.com/ndownloader/files/42480723. (DOCX) [file pone.0297628.s014.docx]

Table S5: Description of Primary outcome and secondary outcomes of Meta-analysis

|  | REMAP-CAP | | ACTIV-4a | | RECOVERY | | ACTIV-4b | | PACT | |
| --- | --- | --- | --- | --- | --- | --- | --- | --- | --- | --- |
| intervention | Pooled antiplatelets | Control | P2Y12 inhibitor | Usual care | Aspirin | Usual care | Aspirin | Placebo | Clopidogrel | No clopidogrel |
| All-cause death, n/total | 299/1011(29.6%) | 170/521(32.6%) | 18/293(6.1%) | 11/269(4.1%) | 1222/7351(16.6%) | 1299/7541(17.2%) | 0/144 | 0/414 | 24/150 | 34/140 |
| Survival to hospital discharge, n/total | 723/1011(71.5%) | 354/521(67.9%) | 275/293(93.9%) | 258/269(95.9%) | 5496/7351(74.8%) | 5548/7541(73.6%) |  |  | 111/150 | 106/140 |
| Any thrombotic event, n/total | 112/996(11.2%) | 65/513(12.7%) | 9/293(3.1%) | 5/269(1.9%) | 339/7290(4.6%) | 396/7457(5.3%) | 0 | 0 | 17/150 | 21/140 |
| Venous thrombotic event, n/total | 87/998(8.7%) | 56/516(10.9%) | 5/293(1.7%) | 5/269(1.9%) | 321/7290(4.4%) | 372/7457(5.0%) | 0 | 0 | 17/150 | 21/140 |
| Arterial thrombotic event, n/total | 37/996(3.7%) | 12/513(2.3%) | 4/293(1.4%) | 1/269(0.4%) | 27/7290(0.4%) | 41/7457(0.5%) | 0 | 0 | 1/150 | 0/140 |
| Pulmonary Embolism，n/total | 72/996(7.2%) | 49/513(9.6%) | 3/293(1.0%) | 4/269(1.5%) | 294/7290(4.0%) | 332/7457(4.4%) | 0 | 0 | 6/150 | 6/140 |
| Myocardial Infarction，n/total | 16/996(1.6%) | 5/513(1.0%) | 3/293(1.0%) | 1/269(0.4%) | 14/7290(0.2%) | 19/7457(0.3%) | 0 | 0 | 0/150 | 0/140 |
| Deep Vein Thrombosis，n/total | 17/996(1.7%) | 8/513(1.6%) | 2/293(0.7%) | 1/269(0.4%) | 27/7290(0.4%) | 40/7457(0.5%) | 0 | 0 | 12/150 | 17/140 |
| Ischemic Cerebrovascular Accident，n/total | 16/996(1.6%) | 4/513(0.8%) | 1/293(0.3%) | 0/269(0%) | 13/7290(0.2%) | 22/7457(0.3%) | 0 | 0 | 0/150 | 0/140 |
| Thrombotic events or death, n/total | 355/1011(35.1%) | 212/521(40.7%) | 18/293(6.1%) | 12/269(4.5%) | - | - | 0 | 0 | 1/150 | 0/140 |
| Major bleeding, n/total | 21/1002(2.1%) | 2/517(0.4%) | 6/293(2.0%) | 2/269(0.7%) | 115/7290(1.6%) | 76/7457(1.0%) | 0 | 0 | 2/150 | 2/140 |
| Organ support–free days, median(IQR) | 7(-1 to 16) | 7(-1 to 16) | 21(20-21) | 21(21-21) | - | - | - | - | - | - |
| Respiratory Support-Free days, median(IQR) | 7(-1 to 16) | 7(-1 to 16) | - | - | - | - | - | - | - | - |
| Vasopressor/Inotrope-Free days, median(IQR) | 19(-1 to 21) | 19(-1 to 21) | - | - | - | - | - | - | - | - |
| Organ support–free days of survivors, median(IQR) | 14(4 to 17) | 14(6.25 to 18) | - | - | - | - | - | - | - | - |
| Alive and free of organ support, n/total | - | - | 218/293(74.4%) | 211/269(78.4%) | - | - | - | - | - | - |
| Alive with organ support, n/total | - | - | 57/293(19.5%) | 47/269(17.5%) | - | - | - | - | - | - |
| Aggravation of illness，n/total | 636/1011 | 326/521 | - | - | 1473/6993(21.1%) | 1569/7169（21.9%） | - | - | - | - |
| Intubation，n/total | 230/636(36.2%) | 133/326(40.8%) | - | - | 772/6993(11%) | 829/7169(12%) | - | - | - | - |
| ECMO( extracorporeal membrane oxygenation) n/total | 4/636(0.6%) | 1/326(0.3%) | - | - | - | - | - | - | - | - |
| Death，n/total | 155/636(24.4%) | 98/326(30.1%) | - | - | 1076/6993(15%) | 1141/7169(16%) | - | - | - | - |
