# Supplementary material for: Effect of antiplatelet therapy after COVID-19 diagnosis: A systematic review with meta-analysis and trial sequential analysis
Source: PLoS One. 2024 Feb 1;19(2):e0297628. doi: 10.1371/journal.pone.0297628 (PMC10833506; doi:10.1371/journal.pone.0297628)
Supplement: S6 Table — https://figshare.com/ndownloader/files/42480768. (DOCX) [file pone.0297628.s015.docx]

Table S6: Description of Primary outcome and secondary outcomes of Meta-analysis for aspirin vs. P2Y12 inhibitor

|  | Aspirin (Critically ill & Non-critically ill) | | | P2Y12 inhibitor (Critically ill & Non-critically ill) | | | |
| --- | --- | --- | --- | --- | --- | --- | --- |
|  | REMAP-CAP  ( Non-critically ill） | REMAP-CAP  ( Critically ill） | RECOVERY | REMAP-CAP  ( Non-critically ill） | REMAP-CAP  ( Critically ill） | ACTIV-4a | PACT ( Critically ill） |
| All-cause death ,n/total | Only OR values are reported without specific values | 165/563 to 170/521 | 1222/7351 to 1299/7541 | Only OR values are reported without specific values | 134/448 to 170/521 | 18/293 to 11/269 | 24/150 to 34/140 |
| Aggravation of illness, n/total | 10/53 to 19/66 | Only merged data are reported. | 1473/6993 to 1569/7169 | 1/23 to 19/66 | Only merged data are reported. | Not reported | Not reported |
| Survival to hospital discharge, n/total | 78/90 to 87/106 | 402/563 to 354/521 | 5496/7351 to 5548/7541 | 63/67to 87/106 | 321/448 to 354/521 | 275/293 to 258/269 | 111/150 to 106/140 |
| Any thrombotic event, n/total | 2/90 to 2/106 | 69/556 to 65/513 | 339/7290 to 396/7457 | 5/67 to 2/106 | 43/440 to 65/513 | 9/293 to 5/269 | 17/150 to 21/140 |
| Venous thrombotic event, n/total | 1/90 to 1/106 | 54/563 to 56/516 | 321/7290 to 372/7457 | 5/67 to 1/106 | 33/448 to 56/516 | 5/293 to 5/269 | 17/150 to 21/140 |
| Arterial thrombotic event, n/total | 1/90 to 1/106 | 22/556 to 12/513 | 27/7290 to 41/7457 | 0/67 to 1/106 | 15/440 to 12/513 | 4/293 to 1/269 | 1/150 to 0/140 |
| Major bleeding, n/total | 1/90 to 0/105 | 11/559 to 2/517 | 115/7290 to 76/7457 | 0/67 to 0/105 | 10/443 to 2/517 | 6/293 to 2/269 | 2/150 to 2/140 |
