# Supplementary material for: Effect of antiplatelet therapy after COVID-19 diagnosis: A systematic review with meta-analysis and trial sequential analysis
Source: PLoS One. 2024 Feb 1;19(2):e0297628. doi: 10.1371/journal.pone.0297628 (PMC10833506; doi:10.1371/journal.pone.0297628)
Supplement: S7 Table — https://figshare.com/ndownloader/files/42480801. (DOCX) [file pone.0297628.s016.docx]

Table S7: Comprehensive overview of included trials

| **Study** | **Study Design** | **Treatment** | **Study Population** | **Inclusion criteria** | **Exclusion criteria** | **Primary Efficacy Endpoint** | **Secondary Efficacy Endpoint** | **Safety Endpoint** |
| --- | --- | --- | --- | --- | --- | --- | --- | --- |
| **REMAP-CAP-2022** | Adaptive platform trial (REMAP-CAP),  a randomized clincial trial,  open label RCT | All antiplatelet interventions were administered enterally un  til study day 14 or hospital discharge, whichever occurred first.  After 14 days, decisions regarding antiplatelet therapy were at  the discretion of treating clinicians. Antiplatelet dosing was as  follows: aspirin, 75 to 100mg once daily; clopidogrel, 75mg once  daily without a loading dose; ticagrelor, 60mg twice daily with  out a loading dose; prasugrel, a 60-mg loading dose followed  by 10mg daily (if aged <75 years and weight ≥60 kg) or 5mg daily  (if aged ≥75 years or weight <60 kg).  As such, only patients randomized to the “no antiplatelet treatment” intervention  within the Antiplatelet Domain served as controls for the analysis of antiplatelet therapeutic efficacy. In  doing so, only concurrent controls were analyzed to determine efficacy of antiplatelet treatment. | 1815 non–critically and critically ill patients hospitalized for COVID-19 | Patients admitted to the hospital, aged 18 years or older, with  clinically suspected or microbiologically confirmed COVID-19  were eligible for enrollment. Patients admitted to an inten  sive care unit (ICU) and receiving respiratory or cardiovascu  lar organ support were classified as critically ill and all others  as non–critically ill. Respiratory organ support was defined as  invasive or noninvasive mechanical ventilation including via  high-flow nasal cannula if the flow rate was at least 30 L/min  and the fraction of inspired oxygen was at least 0.4. Cardio  vascular organ support was defined as receipt of vasopres  sors or inotropes. | Exclusion criteria included presumption that death was imminent with lack of commitment to full support, clinical or laboratory-based bleeding risk sufficient to contraindicate antiplatelet therapy, creatinine clearance less than 30 mL/min or receipt of kidney replacement therapy, enrollment in an external trial of anticoagulation or antiplatelet therapy, or enrollment in the anticoagulation domain of the trial platform for participants older than 75 years. Patients were also excluded if theywere already receiving antiplatelet therapy or nonsteroidal anti-inflammatory drugs (NSAIDs), if a clinical decision had beenmade to commence antiplatelet or NSAID therapy, or if a treating clinician believed that participation in the domain would not be in the best interests of a patient. Critically ill patients had to be enrolled within 48 hours of admission to an ICU. Patients were enrolled from 105 sites in 8 countries (Canada, France, Germany, India, Italy, Nepal, the Netherlands, and the United Kingdom). | The primary outcome was respiratory and cardiovascular  organ support–free days to day 21. In this composite ordinal  outcome, all deaths occurring during the index hospitalization were assigned the worst possible outcome (–1). Among  survivors, respiratory and support-free support–free  days were calculated up to day 21 (survivors with no organ  support were assigned a score of 22). | Secondary outcomes were survival to day 90, progression to invasive mechanical ventilation, extracorporeal membrane oxygenation or death among those not receiving that support at baseline,  vasopressor-/inotrope-free days, respiratory support–free  days, duration of ICU stay, duration of hospital stay, serious adverse events, World Health Organization ordinal score for clinical improvement (ranging from 0 [no evidence of infection] to 8 [death]), major bleeding up to day 14 defined according to International Society of Hemostasis and Thrombosis criteria including fatal and intracranial bleeding, venous thromboembolism (deep vein thrombosis, pulmonary embolism, and other venous thromboembolism), arterial thrombosis (cerebrovascular event, myocardial infarction, and other arterial  thrombotic event), as well as a composite of thrombosis or  death. | major bleeding up to day 14 defined according to International Society of Hemostasis and Thrombosis criteria including fatal and intracranial bleeding, venous thromboembolism (deep vein thrombosis, pulmonary embolism, and other venous thromboembolism), arterial thrombosis (cerebrovascular event, myocardial infarction, and other arterial thrombotic event) |
| **ACTIV-4B 2021** | A minimal-contact, adaptive, randomized, double-blind, placebo-controlled trial | Therapeutic ：aspirin (81 mg once daily)  versus  placebo twice daily for  45 days | 657 symptomatic outpatients  with COVID-19 conducted in the US | --Ambulatory patients between the ages of 40 and 80 years with newly diagnosed symptomatic SARS-CoV-2 infection  -with positive polymerase chain reaction or antigen test results were eligible.  -Creatinine clearance＞30mL/min/1.73m2  -platelet count＞100 000/mm3  -negative pregnancy test for WOCBP  -ability to be contacted by telephone or other electronic methods of communication | -Indication for therapeutic anticoagulation (mechanical heart valve, AF, APS)  -Indication for single or dual antiplatelet therapy  -lactating  -primary brain tumor or acute leukemia  -bleeding risk:  -hospitalization in the past 2 months for:  -bleeding due to ulcer or GI tract disease  -major surgery, stroke, or intracranial hemorrhage  -platelet count < 100,000 per microliter can be obtained after randomization  -calculated creatine clearance < 30 ml/min can be obtained after randomization  -ever hospitalized after diagnosis of COVID-19  -concomitant need for strong inducers/inhibitors of p-gp and CYP3A4 (17: 775  Appendix B)  -SARS-CoV-2 PCR or antigen test more than 14 days prior  -Unable to give written informed consent | The composite of symptomatic deep  venous thrombosis, pulmonary embolism, arterial thromboembolism, myocardial infarction, ischemic stroke, hospitalization for cardiovascular or pulmonary events, and allcause mortality for up to 45 days after treatment initiation. | included the individual components of the primary study end point as well as  mortality without antecedent hospitalization. | major bleeding and clinically relevant nonmajor bleeding  (CRNMB) as defined by International Society on Thrombosis  and Hemostasis (ISTH) criteria, as well as any events of disseminated intravascular coagulation. |
| **RECOVERY 2022** | A individually randomised, controlled, open-label, platform，investigator-initiated trial | Therapeutic ：standard of care plus 150 mg aspirin once per day until discharge or  usual standard of care alone using web based simple (unstratified) randomisation with allocation concealment  versus usual standard of care | 14 892 patients hospitalised with COVID-19 | -Hospitalised  -SARS-CoV-2 infection associated disease (clinically suspected or laboratory confirmed)  - No medical history that might, in the opinion of the attending clinician, put the patient at significant risk if he/she were to participate in the trial | - Patients with known hypersensitivity to aspirin, a recent history of major bleeding, or currently receiving aspirin or another antiplatelet treatment  - Children younger than 18 years？ | -all-cause mortality | -time to discharge from hospital, and, among patients not on invasive mechanical ventilation at andomisation, progression to invasive mechanical ventilation (including extracorporeal membrane oxygenation) or death | use of non-invasive respiratory support, time to successful cessation of invasive mechanical ventilation (defined as cessation of invasive mechanical ventilation within, and survival to, 28 days), use of renal dialysis or haemofiltration, cause-specific mortality, major bleeding events (defined as intracranial bleeding or bleeding  requiring transfusion, endoscopy,surgery, or vasoactive  drugs), thrombotic events (defined as acute pulmonary embolism, deep-vein thrombosis, ischaemic stroke, myocardial infarction, or systemic arterial embolism) and major cardiac arrhythmias. |
| **ACTIV-4a 2021** | An open-label, bayesian, adaptive randomized clinical  Trial | Therapeutic ：a therapeutic dose of heparin plus a P2Y12  inhibitor (n = 293)  versus a therapeutic dose of heparin only (usual care) (n = 269) in a 1:1 ratio | 562 non–critically ill patients hospitalized for COVID-19 | -≥ 18 years of age  -Hospitalized for COVID-19  -Enrolled within 72 hours of hospital admittance or 72 hours of positive COVID test  -Expected to require hospitalization for > 72 hours  -See arm-specific Appendices for additional criteria and details | -Imminent death  -Requirement for chronic mechanical ventilation via tracheostomy prior to hospitalization -Pregnancy  -See arm-specific appendices for additional criteria and details. | -21 Day Organ-Support free-days. The primary endpoint is the number of days that a patient is alive and free of organ support through 21 days after trial entry. （Organ support is defined by receipt of invasive or non-invasive mechanical ventilation, high flow nasal oxygen, vasopressor therapy, or ECMO support. ） | -Key Secondary Endpoint: A composite endpoint of death, pulmonary embolism, systemic arterial thromboembolism, myocardial infarction, or ischemic stroke during hospitalization or at 28 days after enrollment (whichever is earlier)  -Other Secondary Endpoints:  --A composite endpoint of death, deep vein thrombosis, pulmonary embolism, systemic arterial thromboembolism, myocardial infarction, or ischemic stroke during hospitalization or at 28 days after enrollment (whichever is earlier)  --28 Day Hospital free days (non-ICU level patients)  --28 Day Ventilator-Free Days (ICU level patients)  --28 Day Vasopressor-Free Days (ICU level patients)  --28 Day Renal Replacement Free Days  --Hospital readmission within 28 days  --Acute kidney injury as defined by KDIGO criteria  --Deep vein thrombosis  --Pulmonary embolism  --Systemic arterial thrombosis or embolism  --Myocardial infarction  --Ischemic stroke  --Use of extracorporeal membrane oxygenation (ECMO) support  --Mechanical circuit (dialysis or ECMO) thrombosis  --All-cause mortality at 28 days  x Organ support free days at 28 days  x All-cause mortality during initial hospitalization (includes death after 28 days)  x WHO ordinal scale (peak scale over 28 days, scale at 14 days, and proportion with  improvement by at least 2 categories compared to enrollment, at 28 days)  x All-cause mortality at 90 days | -Major Bleeding (as defined by the ISTH)  -Symptomatic intracranial or intracerebral hemorrhage (evaluated as a separate endpoint from other major bleeding)  -Confirmed Heparin induced thrombocytopenia (laboratory confirmed by anti-PF4 test or Serotonin Release Assay (SRA)) |
| **PACT** | open-label,  blinded end point adjudication,  RCT | Clopidogrel 300 mg administered once orally on the day of randomization, followed by  75 mg administered once daily on subsequent days | 190 critically ill patients hospitalized for COVID-19 | Eligible patients were at least 18 years of age with an acute infection with SARS-CoV2 who were requiring intensive care unit (ICU) level of care, were at that level of care for ≤96 hours before randomization, and did not have an indication for full-dose anticoagulation. ICU level of care was defined as (1) being admitted to an ICU or (2) being cared for in a non-ICU room by an ICU team or requiring advanced respiratory support (ie, invasive mechanical ventilation, noninvasive positive pressure ventilation, or high-flow nasal canula for respiratory insufficiency), continuous vasopressor use, or mechanical circulatory support. | Exclusion criteria included any ongoing or planned use of full-dose anticoagulation for any indication, ongoing or planned use of dual antiplatelet therapy, contraindication to antithrombotic therapy, high risk of bleeding (including fibrinogen <200 mg/dL), history of heparin-induced thrombocytopenia, or ischemic stroke within the past 2 weeks. | A hierarchical composite of venous and arterial thrombotic events, defined in the following order: death attributable to venous or arterial thrombosis, pulmonary embolism, clinically evident deep venous thrombosis (DVT), type 1 myocardial infarction, ischemic stroke, systemic embolic event or acute limb ischemia, and clinically silent DVT. | Death attributable to venous or arterial thrombosis, pulmonary embolism, clinically evident deep venous thrombosis (DVT), type 1 myocardial infarction, ischemic stroke and systemic embolic event or acute limb ischemia. | Fatal or life-threatening bleeding, defined as a bleeding event that led to death or was intracranial, intrapericardial with tamponade, associated with hemodynamic instability requiring intervention, or resulted in transfusion of at least 4 units over 24 hours. |
