# Supplementary material for: Effect of antiplatelet therapy after COVID-19 diagnosis: A systematic review with meta-analysis and trial sequential analysis
Source: PLoS One. 2024 Feb 1;19(2):e0297628. doi: 10.1371/journal.pone.0297628 (PMC10833506; doi:10.1371/journal.pone.0297628)
Supplement: S8 Table — https://figshare.com/ndownloader/files/42480810. (DOCX) [file pone.0297628.s017.docx]

Table S8: Quality assessment of included studies by Risk of bias 2.0

| Study | Randomization process | Deviations from intended interventions | Missing outcome data | Measurement of the outcome | Selection of the reported result | Overall Bias |
| --- | --- | --- | --- | --- | --- | --- |
| REMAP-CAP-2022 | Low risk | Low risk | Low risk | Low risk | Low risk | Low risk |
| ACTIV-4a-2021 | Low risk | Low risk | Low risk | Low risk | Low risk | Low risk |
| Recovery-2022 | Low risk | Some concern | Low risk | Low risk | Low risk | Some concern |
| ACTIV-4b-2021 | Low risk | Low risk | Low risk | Low risk | Low risk | Low risk |
| PACT | Low risk | Low risk | Low risk | Low risk | Low risk | Low risk |
