# Supplementary material for: Effect of antiplatelet therapy after COVID-19 diagnosis: A systematic review with meta-analysis and trial sequential analysis
Source: PLoS One. 2024 Feb 1;19(2):e0297628. doi: 10.1371/journal.pone.0297628 (PMC10833506; doi:10.1371/journal.pone.0297628)
Supplement: S9 Table — https://figshare.com/ndownloader/files/42480819. (DOCX) [file pone.0297628.s018.docx]

Table S9: Evidence table for outcome measure

|  | |  | | | |  |  |  |  |  |  |
| --- | --- | --- | --- | --- | --- | --- | --- | --- | --- | --- | --- |
| Outcomes | Risk of Bias* | | Inconsistency† | Indirectness‡ | Imprecision§ | | Other Considerations | Antiplatelet  therapy | Control Groups | Relative(95%CI) | Certainty |
| All-cause death | Not serious | | Not serious | Not serious | Not serious | | None | 8799 | 8745 | RR 0.96, 95% CI, 0.90-1.02 | HIGH |
| Survival to hospital discharge | Not serious | | Not serious | Not serious | Not serious | | None | 8655 | 8331 | RR 1.02, 95% CI, 1.00-1.04 | HIGH |
| Any thrombotic event | Not serious | | Not serious | Not serious | Not serious | | None | 8723 | 8653 | RR 0.89, 95%CI 0.78-1.01 | HIGH |
| Venous thrombotic event | Not serious | | Not serious | Not serious | Not serious | | None | 8724 | 8656 | RR 0.87, 95%CI 0.76-0.99 | HIGH |
| Arterial thrombotic event | Not serious | | Not serious | Not serious | Not serious | | None | 8723 | 8653 | RR 0.98, 95%CI 0.68-1.42 | HIGH |
| Major bleeding | Not serious | | Not serious | Not serious | Not serious | | None | 8729 | 8657 | RR 1.71, 95%CI 1.29-2.25 | HIGH |

*More than 25% of studies with a risk of bias (ie, inappropriate sampling method or statistical analyses).

†More than 25% of studies with small sample size.

‡More than 25% of studies did not use valid and reliable methods for data collection.

§Heterogeneity across the studies (prediction interval has a variation ≥0.5 between upper and lower limits).
